# Posterior Thalamic Nucleus Modulation of Tactile Stimuli Processing in Rat Motor and Primary Somatosensory Cortices

**DOI:** 10.3389/fncir.2017.00069

**Published:** 2017-09-27

**Authors:** Diana Casas-Torremocha, Francisco Clascá, Ángel Núñez

**Affiliations:** Department of Anatomy, Histology and Neuroscience, Faculty of Medicine, Autonomous University of Madrid, Madrid, Spain

**Keywords:** thalamocortical, whiskers, barrel field, electrophsyiology, optogenetics

## Abstract

Rodents move rhythmically their facial whiskers and compute differences between signals predicted and those resulting from the movement to infer information about objects near their head. These computations are carried out by a large network of forebrain structures that includes the thalamus and the primary somatosensory (S1BF) and motor (M1wk) cortices. Spatially and temporally precise mechanorreceptive whisker information reaches the S1BF cortex via the ventroposterior medial thalamic nucleus (VPM). Other whisker-related information may reach both M1wk and S1BF via the axons from the posterior thalamic nucleus (Po). However, Po axons may convey, in addition to direct sensory signals, the dynamic output of computations between whisker signals and descending motor commands. It has been proposed that this input may be relevant for adjusting cortical responses to predicted vs. unpredicted whisker signals, but the effects of Po input on M1wk and S1BF function have not been directly tested or compared *in vivo*. Here, using electrophysiology, optogenetics and pharmacological tools, we compared in adult rats M1wk and S1BF *in vivo* responses in the whisker areas of the motor and primary somatosensory cortices to passive multi-whisker deflection, their dependence on Po activity, and their changes after a brief intense activation of Po axons. We report that the latencies of the first component of tactile-evoked local field potentials in M1wk and S1BF are similar. The evoked potentials decrease markedly in M1wk, but not in S1BF, by injection in Po of the GABA_A_ agonist muscimol. A brief high-frequency electrical stimulation of Po decreases the responsivity of M1wk and S1BF cells to subsequent whisker stimulation. This effect is prevented by the local application of omega-agatoxin, suggesting that it may in part depend on GABA release by fast-spiking parvalbumin (PV)-expressing cortical interneurons. Local optogenetic activation of Po synapses in different cortical layers also diminishes M1wk and S1BF responses. This effect is most pronounced in the superficial layers of both areas, known to be the main source and target of their reciprocal cortico-cortical connections.

## Introduction

Rodents move their whiskers to sense their nearby environment. Extracting sensory information from whisker movement crucially depends on feedback-based, precise comparisons of the intended and resulting motion. These computations are believed to be mainly carried out in the “barrel” field of the primary somatosensory cortex (S1BF) and the whisker region of the primary motor cortex (M1wk; Ahissar and Kleinfeld, 2003; Ferezou et al., 2007). Mechanoreceptive information from hair follicles in the whisker pad may reach these cortices via two main pathways: (a) the lemniscal pathway, that is relayed to S1BF via the ventroposterior thalamic nucleus (VPM); and (b) the paralemniscal pathway, that is relayed to both S1BF and M1wk via the posterior medial thalamic nucleus (Po; reviews in Bosman et al., 2011; Kleinfeld and Deschênes, 2011). In S1BF, VPM axons terminate mainly in layer (L)4, and also at the edge between L5b and L6a, while Po axons target L5a and L1 (Lu and Lin, 1993; Meyer et al., 2010; Wimmer et al., 2010). In M1wk, Po axons terminate mainly in L2/3 (Deschênes et al., 1998; Ohno et al., 2012; Hooks et al., 2015).

Lemniscal VPM neurons are readily activated by passive single-whisker movement and exhibit clear directional tuning, making them ideally suited for coding fine stimulus features. In contrast, paralemniscal Po neurons respond far less reliably to tactile inputs, requiring the stimulation of several adjacent whiskers. There are indications that Po neurons may even change their latencies depending of the stimulation frequency (Ahissar et al., 2000; see however, Masri et al., 2008). Moreover, Po cell responses present lower frequency-adaptation (Moore, 2004) and may be weakly modulated by passive or active whisker movements (Lavallée et al., 2005; Urbain et al., 2015). Overall, these features make Po neurons less well suited for coding with high accuracy the tactile stimulus features (Diamond et al., 1992; Yu et al., 2006). Unlike VPM, Po receives collateral connections from corticofugal layer 5 neurons; thus, Po neurons might perform fast, complex computations between ascending whisker signals and outgoing motor commands, which may be important for the fine analysis and control required to extract information from whisker movement (Ahissar and Zacksenhouse, 2001; Sosnik et al., 2001; Groh et al., 2014; Ahissar and Oram, 2015).

In S1BF, Po input has been shown to monosynaptically excite pyramidal neurons *in vitro* (Petreanu et al., 2009; Audette et al., 2017) and to drive their firing *in vivo* (Gambino et al., 2014; Jouhanneau et al., 2014; Mease et al., 2016). In parallel, Po activation inhibits pyramidal cell firing through a di-synaptic mechanism involving GABAergic interneurons (Castejon et al., 2016; Audette et al., 2017). Early works have characterized the distribution, morphology and abundance of local GABAergic interneuron populations in the cortex based on the expression of the calcium-binding proteins (reviewed in DeFelipe et al., 2013). These interneurons display different discharge patterns and modulate both the responsiveness of individual pyramidal neurons and the spatial-temporal activity patterns of cortical cell populations (Murayama et al., 2009; Kvitsiani et al., 2013; Agetsuma et al., 2017). Fast-spiking parvalbumin (PV) interneurons have been shown to be a synaptic target of thalamocortical axons in other areas (Staiger et al., 1996; Kubota et al., 2007), and, in S1BF their GABAergic activity is believed to be important for sensory processing and for gating transformation of sensory signals into goal-directed motor output (Castejon et al., 2016; Sachidhanandam et al., 2016; Yu et al., 2016).

The effect of Po input on M1wk cell activity is far less well characterized. *In vitro* activation of Po axons produces responses in L2-L5 M1wk pyramidal cells (Hooks et al., 2013, 2015; Yamawaki et al., 2014). Tactile inputs lead to cell firing in M1wk upper layers cells *in vivo*; however, this is assumed to depend mainly on an activation of S1BF and the subsequent excitation of M1wk cells through cortico-cortical connections (Ferezou et al., 2007; Mao et al., 2011). Some authors have noted that response profiles of M1wk cells to different frequencies of tactile stimulation show similarities to the profile responses recorded directly in Po cells (Ahissar et al., 2000; Chakrabarti et al., 2008), and suggested that Po activity may be involved in M1wk responses, either directly or via a relay in S1BF. Nevertheless, the impact of tactile inputs relayed directly to M1wk via Po remains unclear.

High-frequency electrical stimulation of VPM axons reduces subsequent whisker-evoked cortical responses in S1BF; this effect is believed to involve both history-dependent depression of thalamocortical synapses and an engagement of cortical inhibitory neurons (Castro-Alamancos and Oldford, 2002; Khatri et al., 2004; Middleton et al., 2010). Thalamocortical suppression of cortical activity might act as a gain regulator in the processing of some sensory signals reaching the neocortex (Castro-Alamancos and Oldford, 2002). For example, during active exploration with whisker movements, a barrage of sensory signals is likely to reach the somatosensory and motor cortices. In this situation, thalamocortical suppression might be important to prevent system overload and to ease detection of biologically-relevant (unpredicted) signals. Thalamocortical suppression might thus allow the cortex to detect relevant signals over a wider range of system noise. Whether Po axons are able to modulate in a similar way the responsiveness of S1BF or M1wk cells has not been yet investigated.

Using a combination of electrophysiology, optogenetics and pharmacology *in vivo*, here we tested the ability of Po inputs to drive M1wk cell firing and to influence subsequent cortical sensory processing in M1wk and S1BF cortex.

## Materials and Methods

### Animals and Anesthesia Procedures

Experiments were performed on 50 adult Sprague-Dawley rats, of both sexes (39 male and 11 female) weighing 250–300 g. No apparent differences in neuronal responses were noticed between animals of different sex under our experimental conditions. We checked that all animals had normal, intact whisker sets at the beginning of the experiments. The animals were housed under standard colony conditions; food and water were supplied *ad libitum*. All procedures were approved by the Ethics Committee of the Autonoma de Madrid University, in accordance with Council Directive 2010/63 of the European Union. Efforts were made to minimize the number of animals required; for example, recordings were always made in the two cerebral hemispheres of each animal used.

All recording sessions were terminal experiments. The anesthetic applied for the recording experiments was urethane (1.6 g/kg i.p.), which induced deep (stage III–IV; Friedberg et al., 1999) anesthesia. During recording sessions, field potential showed the presence of delta frequency waves (1–4 Hz) of high amplitude (>50 μV). In addition, the level of anesthesia was monitored by the absence of both spontaneous whisker movements and pinch withdrawal reflex. Supplemental doses of urethane (0.5 g/kg i.p.) were administered when required to keep a stable anesthetic level. For the stereotaxic injection of viral vectors in the thalamus, we used ketamine (70 mg/kg, i.p.) + xylazine (5 mg/kg, i.p.) + atropine (0.05 mg/kg, i.p.) for induction, followed by inhalatory isofluorane (0.5%–1% in oxygen) for maintenance. Buprenorphine hydrochloride (0.075 mg/kg, s.c.) was administered for post-surgical analgesia.

Animals were euthanized with an intraperitoneal overdose of sodium pentobarbital (80 mg/kg).

### Electrophysiological Recordings

The animal was placed on a water-heated pad (Gaymar T/Pump, Orchard Park, NY, USA) set at 37°C to keep body temperature stable, and its head positioned in a rodent stereotaxic frame (David Kopf Instruments, Tujunga, CA, USA). After a midline skin incision, the periosteum and muscle were retracted, to expose the skull. Local anesthetic (Lidocaine 1%) was applied to all skin incisions. A craniotomy was then drilled and enlarged using mini-rongeurs, and the duramater over the target areas was opened.

Tungsten macroelectrodes (<1 MΩ) or microelectrodes (2–4 MΩ; World Precision Instruments, WPI; Sarasota, FL, USA) were lowered stereotaxically in order to obtain field potentials and unit recordings, respectively, in whisker motor cortex (M1wk, i.e., the region where minimal intensity stimulation produces selective whisker movements, situated in cytoachitectonic areas AgM/M2 and along its border with AgL/M1 of the frontal cortex; AP: +0.5–2.5 mm, L: 0.2–1 mm; Donoghue and Wise, 1982; Smith and Alloway, 2013) or in the barrel field of S1 (S1BF; AP: −0.5 to −4 mm, L: 4–6 mm), according to the Paxinos and Watson (2007) Atlas. In each animal, cells in M1wk and S1BF sites responding to the stimulation of the same macrovibrissae were recorded. Since the neurons responding to whisker stimulation showed a variable localization within M1wk, we first stimulated different macrovibrissae until finding a site showing a consistent response. We then placed a recording electrode in S1BF in the cortical point representing the same vibrissae.

Recordings were performed in the superficial (D: 200–600 μm) or deep (D: 900–1500 μm) cortical layers. In each experiment, we recorded responses in different cortical depths in both S1BF and M1wk areas. Later, we grouped all responses recorded at depths of 200–600 μm within the “superficial layer” category, and those recorded between 900–1500 μm in the “deep layers” category. In every animal used, we obtained 2–3 superficial layer datasets and 2–3 deep layer datasets. The 300 μm gap between the superficial and deep layers was devised as a safeguard against potential errors in laminar localization due to small misalignments or drift of the recording electrode from the intended cortical depth. The recording signals were filtered (0.3–300 Hz for local field potentials and 0.3–3 kHz for units) and amplified using a DAM80 preamplifier (WPI). These data were recorded continuously, sampled at 1 or 10 kHz, respectively, via an analog-to-digital converter built in to the Power 1401 data acquisition unit, and fed into a PC computer for off-line analysis with Spike2 software (Cambridge Electronic Design, Cambridge, UK).

### Optogenetic Experiments

Optogenetic experiments were carried out to test the effect of selectively activating *in vivo* the Po thalamocortical axon terminals in either M1wk or S1BF. To this end, we first injected the adeno-associated viral vector AAV5-CaMKIIα::ChR2(H134R)-eYFP.WPRE.hGH (Addgene26969P; Penn Vector Core, University of Pennsylvania) into Po. This vector drives high levels of expression of the light-activated cation channel, channelrhodopsin-2, tagged with the enhanced version of the yellow moiety of the *Aqueoria victoria* fluorescent protein (ChR2-eYFP) in the Ca^2+^/calmodulin-dependent protein kinase II α (CaMKIIα)-expressing cells, ensuring that thalamo-cortical cells transport the protein product to the terminal arborizations in the cortex (Aravanis et al., 2007).

We stereotaxically injected this vector into Po, in both cerebral hemispheres in 11 rats (22 hemispheres). Animals were positioned in a David Kopf stereotaxic apparatus and placed on a water-heated pad set to 37°C. The midline of the scalp was sectioned and retracted, and a small craniotomy was drilled over Po (AP: −3.4 mm, L: 2 mm; Paxinos and Watson, 2007), which is far from the areas to be stimulated/recorded in the second part of these experiments. The vector was diluted in phosphate buffer saline (PBS) to a final concentration of 1.6 × 10^11^ particles/ml; 30–100 nl of this suspension were pressure-injected through a borosilicate glass micropipette (outer tip diameter 15–20 μm), attached to a Picospritzer II precision electrovalve system (Parker-Hannifin, Cleveland, OH, USA). The micropipette tip was then left in place for 10 min before removing it from the brain. Finally, the bone defect was covered with a layer of hemostatic sponge (Spongostan^®^, Ferrosan, Soeborg, Denmark) soaked in saline, and the muscle and skin sutured over it. Animals were allowed to recover, and returned to their cages.

Following a 4-week survival period to allow a robust level of ChR2-eYFP expression by Po cells and its transport to their axon terminals in cortex (Wang et al., 2013), we performed the electrophysiological recordings using a light-stimulation protocol. For these experiments, animals were anesthetized in the same conditions described above. After re-opening and retracting the skin, craniotomies were made to expose the surface of the cerebral cortex in S1BF and M1wk in both hemispheres; then, the duramater was incised and retracted, and the cortical surface kept moist with sterile saline irrigation.

### Optical Stimulation

Light-induced depolarization of ChR2-eYFP-expressing axon terminals was achieved using the beam from a light-emitting diode (LED; Thomas Recording, Germany) delivered either through an optical fiber (core diameter 120 μm) or through an optrode, composed of a tungsten macroelectrode 0.5–0.8 MΩ attached to an optical fiber (core diameter 120 μm; Thomas Recording, Germany). To selectively activate the ChR2-eYFP-expressing Po neuron axons, the optical fiber tip or optrode were inserted directly into different layers of M1wk or S1BF using a micromanipulator (WPI).

The LED was triggered with a square-step voltage command. Stimulation consisted of a single long-lasting pulse of light (473 nm; 300 ms, square rise-fall, which was the time needed to reach maximum intensity). Illumination intensity was <30 mW/mm^2^, which is below the damage threshold of ~100 mW/mm^2^ for blue light (Cardin et al., 2010). Under these conditions, the effective area of ChR2 activation is restricted to few hundred microns, since total light energy transmitted within the cortical tissue *in vivo* decreases rapidly with distance (to around 50% by 100 μm and by 90% at 1 mm; Aravanis et al., 2007; Azimipour et al., 2014).

### Electrical Stimulation

Electrical stimulation of Po thalamocortical neurons was applied using 120 μm diameter stainless steel bipolar electrodes (WPI) stereotaxically positioned into the nucleus. Current intensity (20–100 μA) was adjusted in each experimental case to the double of the minimum required to elicit responses in the recorded area. Single square pulses (0.3 ms duration) were applied (Cibertec Stimulator, Madrid, Spain). Pulses were also applied as single trains at 50 Hz for 1 s. We selected these parameters to resemble the effect of the sustained multiple vibrissae stimulation likely to occur during whisking episodes. High-frequency electrical stimulation evokes highly synchronous thalamic firing and facilitates the study of long-lasting cortical effects. As shown by Middleton et al. (2010), sensory transmission in thalamocortical circuits is maintained at stimulation frequencies up to 100 Hz although whisker responses decrease. In addition, thalamocortical neurons are strongly driven by whisker-evoked trigeminothalamic inputs, enabling them to fire in response to high frequency inputs (Deschênes et al., 2003), even with higher rates than the stimulation frequency applied in our experiments.

### Sensory Stimulation

**Figure 1:**
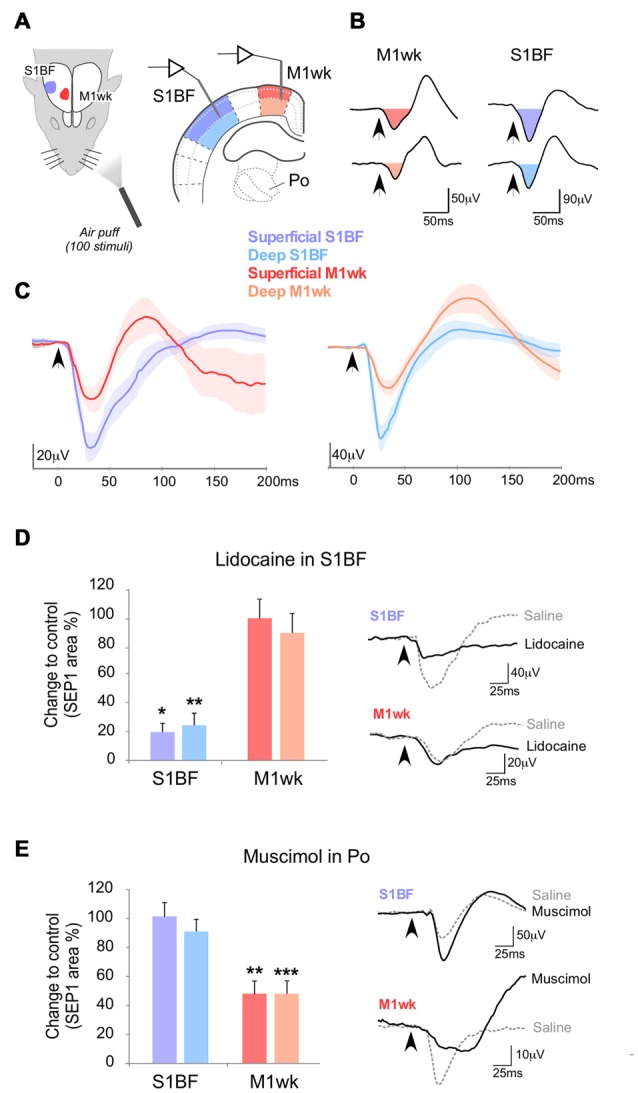
Somatosensory-evoked potentials (SEPs) in M1wk and S1BF cortex by passive multi-whisker stimulation. **(A)** Diagram of the experimental protocol. Tactile stimulation (air puffs) was applied to the trimmed contralateral whiskers (100 stimuli, 1 Hz) of urethane anesthetized rats, and SEPs were recorded in the S1BF (shaded in blue) and M1wk (shaded in red) cortices. In each area, separate recordings were made in the superficial and deep layers (indicated in shades of color). **(B)** Representative SEPs recorded in superficial and deep cortical layers. SEPs consist of negative waves that are followed by positive waves. The color-filled areas indicate somatosensory-evoked field potentials (SEP1), the first component of the evoked potential that was compared between experiments. Arrowheads indicate the moment of whisker stimuli application. **(C)** Overlap of averaged SEP waveforms (*n* = 7 in each case) recorded in superficial and deep layers. Lines indicate mean values and lightly-shaded areas indicate standard errors. **(D)** Effect of S1BF silencing (lidocaine 2% injection, 0.1–0.3 μl) on cortical responses to whisker stimulation. In this and in the following figures, changes in SEP1 area in S1BF and M1wk are shown as the percentage of differences to control values. Representative examples of SEPs recorded in M1wk and S1BF under lidocaine application in S1BF and control condition are shown on the right. **(E)** Effect silencing Po with injected muscimol (1 mM, 0.1 μl) on cortical responses to whisker stimulation. Changes in SEP1 curve areas in S1BF and M1wk are shown as in **(D)**. Representative examples of SEPs recorded in M1wk and S1BF after muscimol and in the control condition are shown on the right. In this and the following figures bars indicate mean and standard errors, *P* < 0.05 (*); *P* < 0.01 (**); *P* < 0.001 (***).

All macrovibrissae were first trimmed to a length of 5 mm. Stereotyped deflections (~15°) of a small group (2–3) posterior whiskers were then produced with air pressure pulses (1–2 kg/cm^2^, 20 ms, released with Picosprizer II electrovalve system) and delivered through a polyethylene tube (1 mm-inner diameter, Figure 1A).

Our control stimulation protocol consisted of a train of 100 air pulses at 1 Hz. In the experimental protocols, identical air pulse trains were applied either three times (spaced by 20 s) following the electrical stimulation of Po (a single train of current pulses at 50 Hz, 1 s), or two times following blue-light activation of Po axonal arborizations in the cortex (a single long-lasting light pulse of 300 ms).

### Pharmacological Modification of the Responses

To gain further insight onto the neuronal circuits involved in M1wk cortex responses to passive whisker movement and their modulation by Po activation, we reduced neuronal activity in either S1BF or Po using locally applied pharmacological agents. In a set of experiments, lidocaine (2% in saline; 0.1–0.3 μl; RBI, Natick, MA, USA) was injected into S1BF cortex to block neuronal activity in this area. In other experiments, a selective agonist for gamma-aminobutyric acid receptor-A (GABA_A_) receptors, muscimol (5-[aminomethyl]-isoxazol-3-ol; 1 mM in saline; 0.1 μl; Sigma-Aldrich, St. Louis, MO, USA) was stereotaxically injected in Po to reduce neuronal activity in the nucleus.

In yet another set of experiments, the P/Q-type voltage-gated calcium channel blocker omega-agatoxin-IVa (1 mM in saline solution; 0.1 μl; Alomone Labs, Jerusalem, Israel), was applied to M1wk. This toxin selectively blocks GABA release in the GABAergic terminals of PV interneuron axons, as well as glutamate in the glutamatergic terminals of some pyramidal neurons (Hefft and Jonas, 2005; Ali and Nelson, 2006; Tottene et al., 2009). The toxin solution was prepared and handled while wearing appropriate protective clothing.

The drugs were slowly delivered through a cannula connected to a Hamilton syringe (1 μl; Bonaduz, Switzerland) over a 1-min period. Recordings started 10 min after drug delivery.

### Perfusion and Histology

After electrophysiological recordings were completed, all animals were euthanized (see above for details) and their brains were serially sectioned for histological verification of the stimulation and recording sites. Though a cannula inserted in the left ventricle, 100 ml of saline followed by 500 ml of 4% paraformaldehyde in 0.1 M phosphate buffer (PB, pH 7.4) were infused. The brains were then stereotaxically blocked and removed from the skull, postfixed for 24 h at 4°C in the same fixative and cryoprotected in a 30% solution of sucrose in PB during for 2–3 days at 4°C. Brains were serially sectioned (50 μm thickness) in the coronal plane on a freezing microtome (Leica SM 2400; Leica Microsystems AG, Wetzlar, Germany).

In the brains of the animals tested with electrical stimulation, two serial sections were collected. They were alternately stained with Nissl or cytochrome oxidase histochemistry (Wong-Riley, 1979) for delineation of cortical layers and thalamic nuclei.

In the brains from the 11 animals tested with optogenetic methods, we examined, in addition, the actual location of the transfected cell somata in the thalamus and terminal axons in the cerebral cortex. To this end, three alternating series of 50 μm sections were collected. The first was mounted for direct eYFP fluorescence visualization. In the second series, eYFP labeling was immunohistochemically enhanced, stabilized and made opaque. On two experiments, we used the third series to perform a double-immunofluorescence protocol against eYFP and PV (details below).

Sections were mounted on glass slides, dehydrated through passage in ascending grades of alcohol, defatted in xylene for 30–60 min and finally coverslipped with DePeX (Serva, Heidelberg, Germany).

### Single Inmunohistochemistry Against eYFP

Following rinses in hydrogen peroxide (2% in PB, 15 min) and Triton X-100 (2% in PB, 30 min), sections were incubated in a purified rabbit antiserum against GFP that also recognizes eYFP (1:500; EXBIO, Prague, Czech Republic) + 2% Triton X-100 + 3% normal goat serum (NGS) + 1% bovine serum albumin (BSA) in PB (16 h, 20°C). After several rinses in PB, sections were incubated in biotinylated goat anti-rabbit IgG (1:100; Sigma–Aldrich, St. Louis, MO, USA) + 2% Triton X-100 + 3% NGS + 1% BSA in PB (2 h, 20°C). Following new rinses in PB, the sections were incubated in avidin-biotin-peroxidase complex solution (1:100; Vectastain Elite, Vector Laboratories, Burlingame, CA, USA) + 2% Triton X-100 (4°C, 16 h). Peroxidase activity was visualized using a glucose oxidase-DAB-nickel protocol (Shu et al., 1988). Sections, were lightly counterstained with Thionin for cytoarchitectonic reference, defattened, dehydrated and coverslippped with DePeX.

### Double eYFP and PV Immunofluorescence

Sections were incubated, free-floating, in the anti-eYFP antiserum (1:500) + purified mouse anti-PV antiserum (1:1500; Swant, Bellinzona, Switzerland) + 2% Triton X-100 + 2% normal donkey serum (NDS) in PB (16 h, 20°C). Following repeated rinses in PB, the sections were then incubated simultaneously with secondary AlexaFluor 488-conjugated anti-rabbit donkey IgG (1:200; Molecular Probes, Carlsbad, CA, USA), and secondary AlexaFluor 647-conjugated anti-mouse donkey IgG (1:200; Molecular Probes) in PB containing 2% Triton X-100 and 2% NDS (2 h, 20°C).

### Electrophysiological Data Analysis

Somatosensory-evoked potentials (SEPs) were extracted in the M1wk and S1BF cortices by calculating the average from responses to 100 whisker stimuli. The mean of the peak latency, peak amplitude and the curve area were calculated from the evoked potentials. The area was measured from the negative slope beginning to the same voltage level with positive slope of the first negative wave. Response magnitude variation of area SEP (%) was calculated using as control (100%) the same stimulation protocol in which Po was not stimulated.

Single-unit activity was extracted with the aid of Spike2 software (Cambridge Electronic Design, Cambridge, UK) for spike waveform identification and analysis. The sorted spikes were stored at a 1-ms resolution and isolated single-units were analyzed and quantified. The mean tactile response was measured from the peristimulus time histogram (PSTH; 1-ms bin width; 100 stimuli) as the number of spikes evoked in the 0–50-ms time window after the stimulus onset divided by the number of stimuli. Neuronal responses larger than two times the mean activity in the 0–50 ms time window before the stimulus onset were considered in this study. The PSTH was calculated from groups of 100 air-puff stimuli in control condition, or after Po thalamocortical pathway stimulation (electrical or optogenetic stimulation). We measured latencies as the time elapsed between stimulus onset and the largest peak in the PSTH. Unitary response magnitude variation (%) was calculated using as control (100%) the first whisker stimulation train before Po stimulation.

Statistical analysis was computed using GraphPad Prism 5 software (San Diego, CA, USA). For single comparisons and normally distributed data (the Shapiro-Wilk normality test), we used the paired Student’s two-tailed *t*-test (STT). Non-normally distributed data were compared with Wilcoxon-matched pairs test (WT) or with Mann-Whitney *U* test (M-WUT) for independent samples. For multiple comparisons after electrical stimulation or blue-light channelrodopsin activation, we used one-way analysis of variance (ANOVA) plus T3 Dunnet’s multiple comparisons as a *post hoc* test. Data are presented as mean ± standard error of the mean (SEM). The threshold level of significance was set at *P* < 0.05, indicating this as (*), *P* < 0.01 as (**) and *P* < 0.001 as (***).

### Anatomical Verification of Recording and Stimulation Sites

Sections stained with Nissl cytochrome oxidase or immunostained for eYFP with the DAB-nickel method were examined under bright-field optics at 10× using a Nikon Eclipse E600 microscope to delineate the precise location of the electrode tracks and vector deposits. Only cases with correct electrode positions in Po were included in the electrophysiological data analysis. Likewise only cerebral hemispheres in which the ChR2-eYFP transfected neurons were confined to Po (sparing the ventrolateral or the ventroposterior medial nuclei according to Paxinos and Watson, 2007 atlas delineation) were included in the optogenetic stimulation data analysis.

Fields containing labeled terminals in M1wk and S1BF were examined at 20×–40×. Images were acquired using a digital camera (DMX 1200F, Nikon, Tokyo, Japan), and panoramic, high-resolution mosaic images were produced using a precision motorized stage (Prior Scientific Instruments, Cambridge, UK) driven by the AnalySIS image software package (Soft Imaging Systems, Münster, Germany). Images illustrated in the figures were adjusted in brightness and contrast using Canvas X software (ACD Systems, Saanichton, BC, Canada).

Double-labeling analysis of eYFP and PV was carried using a Spectral Leica TCS SP5 confocal microscope by sequentially applying argon (488 nm) and helium–neon (543 nm) laser lines to ensure complete channel separation. Image stacks were obtained moving the sample in the Z-axis in increments of 0.5 μm. Regions of interest were imaged using a 63× oil objective (plus 3× digital zoom). Image stacks and maximal projections were analyzed both in separate and merged channels. Orthogonal views were generated using ImageJ open access software (National Institute of Mental Health, Bethesda, USA).

## Results

### Somatosensory-Evoked Field Potentials in S1BF and M1wk have Similar Latencies

The field potentials evoked by the application of air puffs to a small zone of the whisker pad (2–3 principal whiskers; SEPs) were recorded separately in the contralateral M1wk and S1BF (Figure 1A). In both M1wk and S1BF, the SEPs elicited by whisker stimulation at 1 Hz consisted of a negative wave followed by a positive wave (Figures 1B,C). We focused our analysis on the first negative component of the SEPs (SEP1) for statistical comparison since it likely reflects the synaptic events directly related to the arrival of the thalamic volley to the cortex. Accordingly, SEP1 latency and duration were far more consistent than the following components of the evoked potentials (shaded in color areas in Figures 1B,C).

Mean SEP1 peak latencies of this first component were only slightly shorter in S1BF (superficial layers 42.6 ± 2.0 ms and deep layers 41.3 ± 1.9 ms; *n* = 14) than with M1wk (superficial layers 43.7 ± 2.1 ms; *n* = 15, and deep layers 47.4 ± 1.7 ms; *n* = 15). This difference is shown in overlapped SEP1s in Figure 1C. Note that in the superficial layers of S1BF and M1wk SEP1 peaks are close (STT, *P* = 0.684), while SEP1 peak in the deep layers of M1wk is delayed compared to that in the deep layers of S1BF (STT, *P* = 0.016).

The SEP1 peak amplitude evoked by whisker pad stimulation, was slightly larger in the superficial layers of both areas than in their deep layers, although these differences were not statistically significant (M1wk: 45.6 ± 5.3 μV in superficial layers vs. 34.1 ± 4.8 μV in deep layers; *n* = 17; M-WUT, *P* = 0.122; S1BF: 185.6 ± 8.3 μV in superficial vs. 169.0 ± 12.5 μV deep layers; *n* = 14; M-WUT, *P* = 0.389).

### The First Component of SEP1 in M1wk Does Not Depend on S1BF Activity

The observation that SEP1 latencies in the superficial layers of S1BF are only marginally shorter (~1 ms) than in M1wk, raised the question whether the whisker pad inputs driving M1wk SEP1 reach this area via a relay in S1BF, or through a direct ascending pathway. It is well established that S1BF projects heavily to M1wk. Thus, the whisker pad inputs driving M1wk might reach this area via a relay in S1BF but also through a direct ascending pathway from the Po nucleus. To investigate this latter possibility, we applied lidocaine in S1BF cortex to reduce its activity, particularly that of its superficial layers, which originate most of the cortico-cortical projections to M1wk (Hooks et al., 2013; 0.1–0.3 μl; 2% in saline solution; Figure 1D) and quantified the changes in response amplitude. Under this condition, as expected, SEP1 area decreased markedly in S1BF (superficial layers: 19.6 ± 6.0% of the initial response, WT, *P* = 0.018; *n* = 8; deep layers: 24.3 ± 8.3% of the initial response; WT, *P* = 0.007; *n* = 8). In contrast, SEP1 area in the M1wk remained essentially unchanged (superficial layers: 98.1 ± 12.6% of the initial response, WT, *P* = 0.612, *n* = 8; deep layers: 88.3 ± 13.3% of the initial response, WT, *P* = 0.315, *n* = 8), indicating that the first component of the SEP in M1wk does not require S1BF cortex activity.

### Thalamocortical Po Neurons May Mediate the First Component of the Field Potential Evoked in M1wk by Whisker Pad Stimulation

Neurons in the thalamic Po nucleus receive direct ascending inputs from the trigeminal complex, respond to passive whisker movement and directly innervate the M1wk cortex (Williams et al., 1994; Ahissar et al., 2000; Ohno et al., 2012). To test if M1wk SEP1 depends on Po activity, we injected into Po through a cannula the GABA_A_ receptor agonist muscimol (0.1 μl; 1 mM) to reduce neuronal firing (Figure 1E). Ten minutes later, we analyzed the effect on SEPs recorded in M1wk and S1BF. Increased Po inhibition reduced SEP1 in M1wk to a half of its initial area (superficial layers: to 48.2 ± 8.0% WT, *P* = 0.001; deep layers: to 48.3 ± 7.1%, *P* < 0.001; *n* = 7 in each layer). In contrast, SEP1 in S1BF was not affected by increased Po inhibition (101.1 ± 8.4% and 91.5 ± 7.8% of the original response in superficial and deep layers, WT, *P* = 0.444 and *P* = 0.230, respectively; *n* = 7 in each layer). The preservation of whisker-evoked responses in S1BF is consistent with the fact that, unlike M1wk this area receives ascending whisker pad inputs from Po as well as from the VPM, which was spared by the muscimol injections. As an additional control, we confirmed that the dose injected abolished responses recorded in Po to whisker stimulation but preserved normal responsivity in the rostrally-adjacent ventral lateral nucleus to motor cortex stimulation (Supplementary Figure S1).

Overall, the above observations are consistent with the notion that tactile whisker pad input is able to drive cell firing in M1wk via Po thalamocortical cells.

### Electric Stimulation of the Po Nucleus Modifies M1wk and S1BF Responses to Subsequent Whisker Pad Stimulation

**Figure 2:**
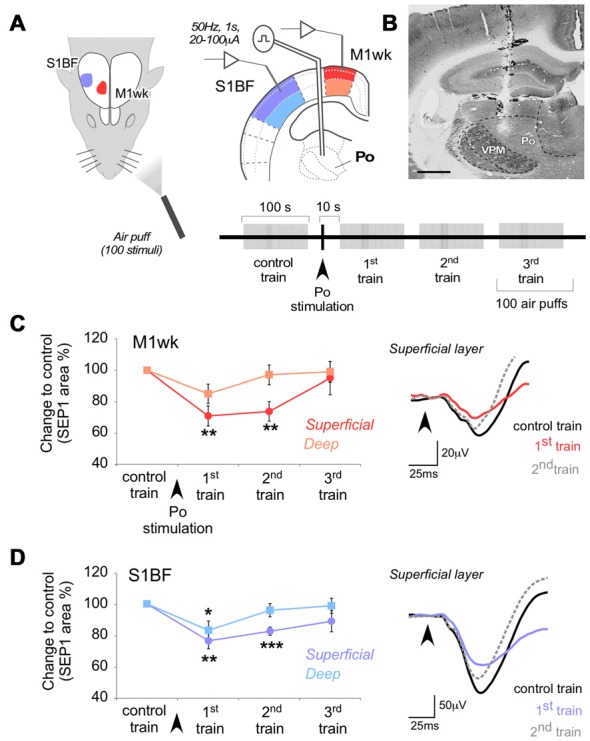
Po stimulation modulates subsequent responses in M1wk and S1BF to passive multi-whisker stimulation. **(A)** Diagram of the experimental protocol. Tactile stimulation (air puffs) was applied to the contralateral whiskers (four trains of 100 stimuli, 1 Hz). A single train of electrical pulses (50 Hz, 1 s, 20–100 μA) was applied to Po immediately after the first train of air puffs (“control train”). SEPs were recorded in the S1BF and M1wk cortices. In each area, separate recordings were made in the superficial and deep layers (shaded in different color hues, respectively). **(B)** Representative image of a coronal brain section counterstained with cytochrome oxidase showing the location of the bipolar electrode tip in Po. Bar = 500 μm. **(C)** Effect of Po electrical stimulation on tactile responses in M1wk cortex. Left: changes in SEP1 area are shown as the percentage of differences to control values. Right: representative individual SEPs recorded during control (black line), first (colored line) and second (gray dashed line) train. Arrowheads indicate whisker stimuli application. **(D)** Effect of Po stimulation on tactile responses in S1BF cortex. Conventions as in **(C)**.

To test the ability thalamocortical Po input to modify the global neuronal activity of M1wk and/or S1BF cortices, we compared the responses in these cortices to tactile whisker pad stimulation before and after a brief electrical stimulation of Po. Specifically, we compared responses to a control train of air-puffs (100 puffs at 1 Hz) delivered before Po stimulation with those produced by identical air-puff trains applied 10–110 s (“1st train”), 120–220 s (“2nd train”) and 230–330 s (“3rd train”) after the application of electrical stimuli (20–100 μA square pulses at 50 Hz, 1 s delivered into Po through a bipolar electrode (Figures 2A,B). We did not observe changes in cortical responses when we delivered a similar protocol without electrical stimulus application (*P* > 0.05 in superficial and deep layers of M1wk and S1BF; data not shown).

Electric Po stimulation produced a decrease in M1wk cortex SEP1 compared to control (*F* = 5.052, *P* = 0.030; Figure 2C). The effect was most pronounced and lasting in the superficial cortical layers (71.1 ± 6.4% of control in the 1st air puff train after Po stimulation, *P* = 0.002; *n* = 18), returning to control levels only in the 3rd air-puff train. In the deep layers of M1wk, although SEP1 amplitude was also reduced, here the effect was smaller and briefer (85.3 ± 6.1% of control in the 1st air-puff train after Po stimulation) and differences were not significant (*F* = 1.639, *P* = 0.148; *n* = 18). Differences in peak latency in superficial or deep M1wk layers after Po activation were also non-significant (WT, *P* = 0.605 and *P* = 0.182, respectively; *n* = 18 in each layer).

Confirming recent observations by Castejon et al. (2016), we observed a depressing effect of electrical Po activation on S1BF cortex responses to whisker pad stimulation (*F* = 6.088, *P* = 0.001 in superficial layers; *F* = 3.252, *P* = 0.028 in deep layers; Figure 2D). Compared to the effect on M1wk responses, the decrease in S1BF SEP1 was of similar amplitude and persistence (superficial layers: 76.4 ± 5.2% of control in the 1st air puff train after Po stimulation, STT, *P* = 0.002, *n* = 16, return to normal in the 3rd train; Deep layers: 83.1 ± 5.9% 1st train after Po stimulation, STT, *P* = 0.041; *n* = 16, return to normal by the time of application of the 2nd train). Peak latencies also increased after Po activation in the superficial S1BF layers (from 43.4 ± 3.0 ms to 44.9 ± 3.2 ms, WT, *P* = 0.386; *n* = 16), but this increase reached statistical significance only in the deep S1BF layers (from 40.9 ± 3.4 ms to 44.4 ± 3.8 ms, WT, *P* = 0.014; *n* = 16).

Taken together, the above observations show that in M1wk, as in S1BF, the repeated activation of Po thalamocortical synapses produces a protracted decrease of the cortical response to subsequent sensory stimulation.

### The Effects of Electric Po Stimulation on M1wk Likely Involve PV-Interneurons

Because fast-spiking PV-expressing GABAergic interneurons control cortical excitability and are a major synaptic target of thalamocortical axons (Cruikshank et al., 2007; Courtin et al., 2013; Zhang et al., 2015) we decided to examine the participation of PV interneurons in the SEP1 reduction in M1wk elicited by Po electrical stimulation. As a selective pharmacological tool to interfere with PV interneuron function, we used omega-agatoxin, a selective blocker of Cav2.1 (P/Q-type) voltage-gated calcium channels. These channels are expressed on PV interneuron axon terminals and mediate their GABA release (Toledo-Rodriguez et al., 2004; Hefft and Jonas, 2005; Zaitsev et al., 2007; Rossignol et al., 2013), although these channels may be also involved in glutamate release from some pyramidal cells (Ali and Nelson, 2006; Tottene et al., 2009).

**Figure 3:**
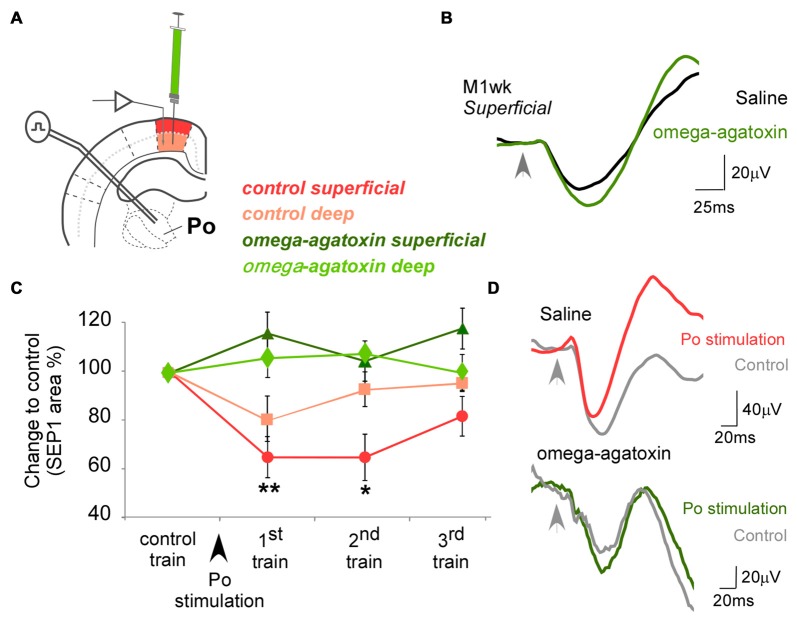
Effect of omega-agatoxin on the modulation exerted by Po on M1wk responsiveness. **(A)** Diagram of the experimental protocol. omega-agatoxin were injected intracortically in M1wk (1 mM; 0.1 μl). **(B)** Case examples of SEPs recorded in superficial layer of M1wk in control condition (black line) and under omega-agatoxin (green line). **(C)** Changes in SEP1 curve areas to whisker stimulation before or after omega-agatoxin injection in M1wk are represented in red or green shades, respectively. Note that Po-evoked inhibition was blocked. **(D)** Case examples of SEPs recorded in superficial layer of M1wk in control condition (upper traces) and under omega-agatoxin (lower traces). SEPs of whisker stimuli control train (gray lines) and the first train after Po stimulation in omega-agatoxin (green line; top) or saline condition (red line; bottom). Arrowheads indicate whisker stimuli application.

Compared to the saline-injected condition, successive trains of air-puff stimulation applied 10 min after injecting of omega-agatoxin into M1wk (1 mM in saline solution; 0.1 μl; Figures 3A,B) had higher SEP1 amplitudes in both superficial (118.0 ± 3.5% compared to control; WT, *P* = 0.012; *n* = 9) and deep cortical layers (114.2 ± 7.1%; WT, *P* = 0.012; *n* = 9). Under omega-agatoxin, Po electrical stimulation did not reduce SEP1 area in either superficial M1wk layers (116.2 ± 7.2% of control, *F* = 2.519, *P* = 0.076; *n* = 9) or deep layers (106.0 ± 10.1%; *F* = 0.016, *P* = 0.079; *n* = 9; Figures 3C,D). In contrast, consistent with our previous experiments, Po stimulation in the saline-injected condition elicited a robust and lasting decrease in superficial layer SEP1 amplitude (64.9 ± 8.5%, *F* = 6.237, *P* = 0.002).

These results suggest that PV-interneurons might be involved in the reduction in M1wk responses elicited by the brief and intense activation of thalamocortical Po neurons.

### Changes in M1wk Unit Activity Elicited by Electrical Po Stimulation

We also recorded single-cell spiking in superficial and deep M1wk neurons and compared this activity between: (a) the non-stimulated (spontaneous) condition; (b) during a simple air-puff stimulation on the whisker pad; (c) after a single pulse of electrical Po stimulation; and (d) during three sequential air-puff trains delivered following a single electrical stimulation train of Po.

**Figure 4:**
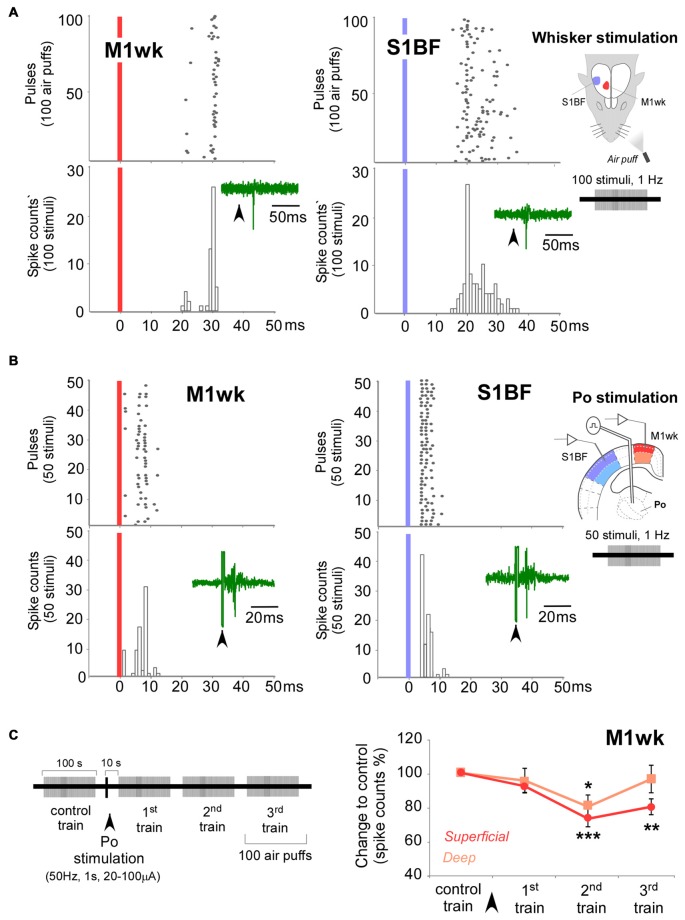
Control exerted by Po nucleus on unit responses in M1wk cortex to passive multi-whisker stimulation. **(A)** Unit responses to whisker stimulation in a representative M1wk and S1BF (for comparison) superficial neuron. Peristimulus time histogram (PSTH) and raster plots shows responses as spike counts during 100 air puffs to the whiskers (1 Hz). Vertical colored bars indicate stimuli application. Representative traces of spikes are shown inside PSTHs. Diagram of the experimental protocol is shown on the right. **(B)** Responses to Po electrical stimulation in a representative M1wk and S1BF (for comparison) superficial neuron. PSTH and raster plots shows responses as spike counts during 50 single-pulse electrical stimulations of Po (1 Hz, 20–100 μA). Conventions as in panel **(A)**. **(C)** Po stimulation modulates whisker unit responses in M1wk. Stimulation protocol is shown in on the left. A single train of electrical pulses (50 Hz, 1 s, 20–100 μA) was applied to Po immediately after the “control train” of whisker pad stimuli. Unit responses were recorded in superficial and deep layers (shaded in darker and lighter color, respectively). Changes in spikes counts are measured as the percentage of differences to control values.

Unit recordings revealed a low spontaneous activity in both superficial and deep cortical M1wk layer neurons (0.5 ± 0.1 spikes/s, *n* = 23; and 0.3 ± 0.1 spikes/s; *n* = 16, respectively). Simple air-puff stimulation of the whisker pad increased spiking in both superficial (1.3 ± 0.1 spikes/stimulus; 23.0 ± 1.8 ms latency to the PSTH peak; *n* = 23) and deep layer cells (0.7 ± 0.02 spikes/stimulus; 24.4 ± 1.2 ms latency; *n* = 16) of M1wk cortex (Figure 4A). We did not find differences between the response times of cells in superficial and deep layers (M-WUT, *P* = 0.689). Electrical stimulation of Po with single pulses (1 Hz, 20–100 μA) induced an orthodromic spike response in both superficial and deep M1wk layer cells (0.7 ± 0.1 spikes/stimulus; 7.8 ± 1.0 ms and 0.7 ± 0.1 spikes/stimulus; 7.3 ± 1.0 ms latency to the PSTH peak, for superficial and deep layers, respectively; Figure 4B), without significant time differences between layers (STT, *P* = 0.742). While all neurons recorded in M1wk responded to air-puff stimulation, we found that only 72% in superficial layers and 64% in deep layers of recorded neurons fired in response to the electrical activation of Po neurons.

Following a single electrical stimulation train of Po, M1wk unit activity was diminished during the 1st air-puff train (10–110 s after Po stimulus, 0.5 ± 0.1 spikes/s to 0.4 ± 0.1 spikes/s; STT, *P* = 0.126; *n* = 8), and then more markedly during subsequent air puff trains, consistent with our previous SEP1 analysis (*F* = 10.919, *P* < 0.001). Specifically, superficial M1wk layer cells lowered their spiking to 92.3 ± 3.6% during the 1st air puff train (*P* = 0.194; *n* = 20), and kept a lowered responsivity during the 2nd (73.4 ± 4.7%; *P* < 0.001) and 3rd (80.4 ± 4.6%; *P* = 0.001) air-puff trains (Figure 4C). In contrast, the spike count decrease in M1wk deep layer neurons was shallower, reaching statistical significance only during the 2nd air puff train, 120–220 s after Po stimulation (80.4 ± 6.8%, *P* = 0.023; *n* = 16), and was back to normal by the 3rd air-puff train.

### *In Vivo* Optogenetic Activation of Thalamocortical Po Synapses Has Different Effects on Whisker Input Processing in M1wk and S1BF

**Figure 5:**
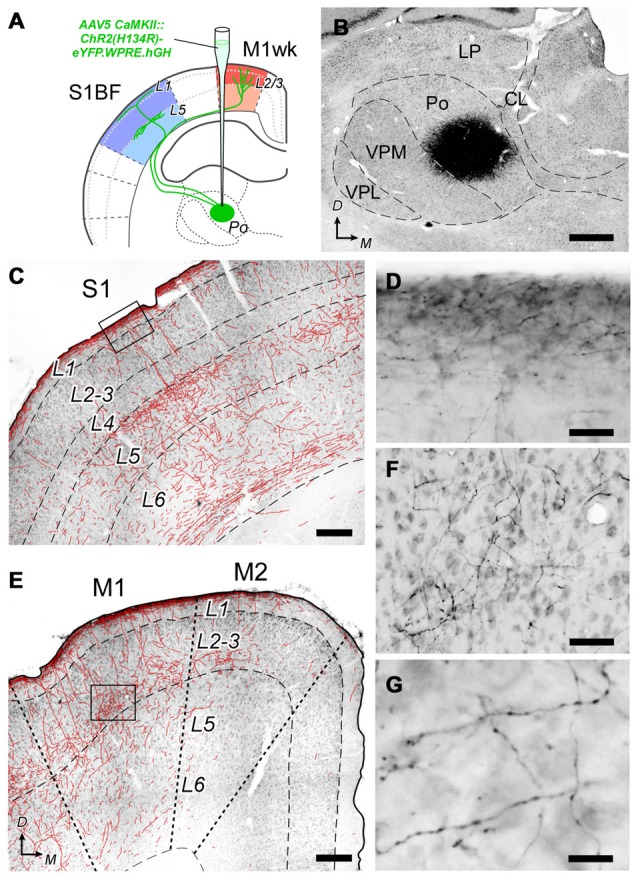
Histological verification of the AAV5 CaMKII::ChR2(H134R) -eYFP.WPRE.hGH vector expression specificity. **(A)** Diagram summarizing the experimental procedure. A suspension of vector particles was stereotaxically injected in Po to drive ChR2 + eYFP expression in a population of the thalamocortical cells innervating M1wk and S1BF. **(B)** The center of a representative vector injection site is shown on a coronal section of the thalamus (the complete series of sections containing the transfected cell somata is illustrated in Supplementary Figure S2). Nickel-enhanced glucose-oxidase immunohistochemistry against eYFP, and light thionin counterstain. Note the “halo” of labeled dendrites radiating around the dense core region that contains the transfected cell bodies. **(C)** Coronal section of the cerebral cortex in S1 showing the distribution of transfected Po axons. To increase axon visibility at this magnification, a camera-lucida line drawing of the axons is overlaid on the image. **(D)** High-magnification detail of labeled axons in L1 of S1 (inset in panel **C**). **(E)** Axonal labeling in the motor cortex. Conventions as in panel **(C)**. **(F)** High-magnification detail of axons labeled in L3 of M1 (inset in panel **E**). **(G)** Axonal varicosities, suggestive of synaptic boutons in L3 of M1. Bars: **B** = 500 μm; **C,E** = 250 μm; **D,F** = 50 μm; **G** = 15 μm. Others abbreviations: CL, Central lateral thalamic nucleus; LP, Lateral posterior thalamic nucleus; VPL, Ventral posterolateral thalamic nucleus.

The above results suggest that activation of Po thalamocortical synapses has different impact on whisker pad input processing in M1wk and S1BF cortices. To directly demonstrate this hypothesis, we decided to use optogenetic activation of Po axon terminals in these cortical areas. We selectively drove the expression of high levels of channelrhodopsin-2, tagged with the fluorescent protein eYFP (ChR2-eYFP) in Po axons innervating M1wk and S1BF cortices by means of stereotaxically-guided injections of adenoviral vectors in this thalamic nucleus (Figure 5A, see “Materials and Methods” Section). To allow protein expression and transport to the axon terminals, recordings were performed 4 weeks post-injection. A blue light pulse (300 ms, square rise-fall) able to stimulate a small volume of tissue (about 100–200 μm in radius; Aravanis et al., 2007; Azimipour et al., 2014) was delivered to the cortex through a thin optic fiber placed in the cortex, pointing downwards. Most of the channel activation produced by the light pulse is likely to have occurred in transfected Po axons located in the ~200 microns immediately below the optic fiber tip (Aravanis et al., 2007, Azimipour et al., 2014, see “Materials and Methods” Section); however, we cannot exclude some weaker additional activation in axon branches located deeper into the cortex. Using this experimental setup, we tested the effect of optogenetic activation of Po synapses on the SEPs and unit responses evoked by whisker pad stimulation in different layers of M1wk and S1BF cortices.

At the end of the recording sessions, animals were sacrificed and perfusion-fixed, and their brains processed for microscope analysis. As the viral vector we used drives the co-expression of ChR2 and eYFP, we ascertained the selective transfection of Po cells and their axons by immunolabeling the brain sections for eYFP. A dense population of neuronal cell somata and dendrites was observed at the injection site in the thalamus (Figure 5B). In 15 out of 22 cerebral hemispheres, this population was limited to the Po nucleus, without encroaching into the adjacent ventral posterior (VP) or ventral lateral (VL) thalamic nuclei; only these experiments were included in the subsequent optogenetic data analysis (see also Supplementary Figure S1).

Labeled Po axons were visible along their path in the white matter and terminal fields in the cerebral cortex. In S1BF, axonal arborizations were most profuse in L5a and L1a (Figures 5C,D). In M1wk, axon branches were most profuse in L3, including its lowest tier, recently identified as the equivalent of L4 in M1 (Yamawaki et al., 2014), as well as in L1a (Figures 5E,F). This laminar pattern is consistent with previous anatomical tracing studies of rat Po axons (Deschênes et al., 1998; Ohno et al., 2012), confirming the selectivity of Po cell transfection. The labeled axons showed numerous varicosities, a direct correlate of synaptic sites in thalamocortical axons (Rodriguez-Moreno et al., 2017; Figure 5G).

### Effect of Optogenetic Po Axons Activation on Sensory Responses in the Same Layer

**Figure 6:**
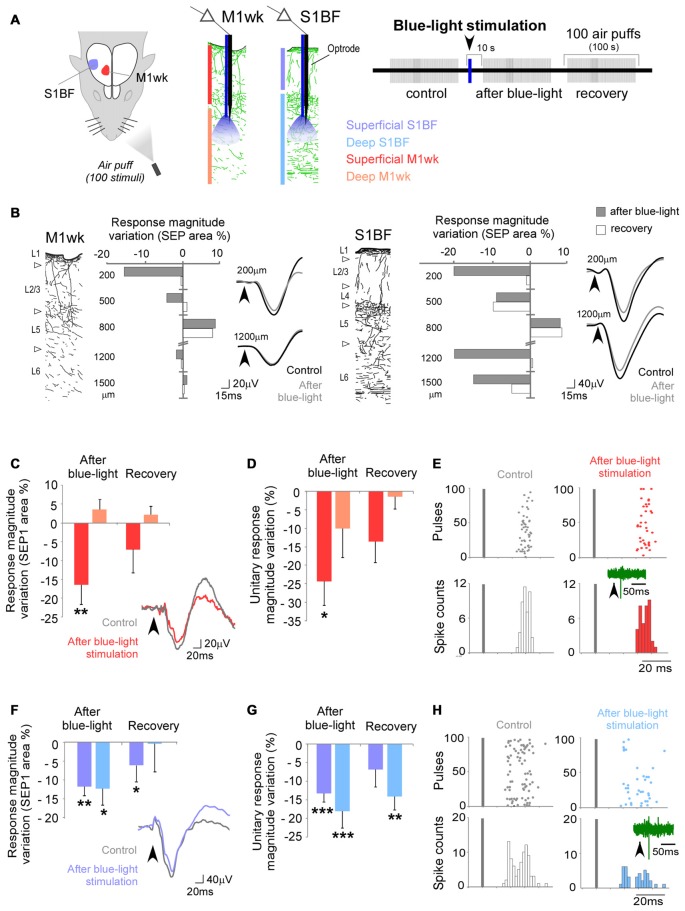
Effects of selective optogenetic stimulation of Po terminal axons in particular layers of M1wk and S1BF cortices. **(A)** Diagram of the experimental protocol. A single pulse of blue-light (473 nm, 300 ms, <30 mW/mm^2^) was applied thought an optrode in the cortex to activate Po axonal terminals expressing ChR2 + eYFP. This blue-light pulse was applied immediately after the “control train” of whisker stimuli. SEPs and unit responses were recorded in the superficial and deep layers (shaded in different color hues, respectively) of S1BF (blue) and M1wk (red) cortices. **(B)** A representative case of these experiments displaying a different laminar innervation pattern and Po effects on tactile responses in M1wk and S1BF cortices. Cortical columns with immunolabeled Po axons are drawn for each area. Changes in SEP1 area immediately after blue-light application (gray) and during the recovery train (second train after Po stimulation; white) are represented as the variation of percentage to the control values in different cortical depths. Negative values symbolize inhibition and positive values facilitation. Representative trace examples of SEPs recorded during control (black line) and after blue-light (gray dashed line) pulses are shown on the right. **(C)** Blue-light activation of Po axons modulates SEP1 in M1wk. Mean values of changes in SEP1 curves area are represented as the variation of percentage to the control values. Examples of SEPs during the control (gray line) and after blue-light application (red line) are shown on the right. Grey bar indicates whisker stimuli application. **(D)** Mean values of changes in unit responses are represented as the variation of spike counts percentage to the control train. **(E)** Examples of PSTH and raster plots during the control (white) and after blue-light application (red) in superficial layers are shown. Representative spike traces are shown inside PSTH. Bars indicate whisker stimuli. **(F)** Blue-light activation of Po axons also modulates SEP1 in S1BF. Conventions as in panel **(C)**, except the example of SEPs after blue-light application is shown as blue line. **(G)** Unit whisker responses in S1BF. Conventions as in panel **(D)**. **(H)** Examples of PSTHs and raster plot of S1BF responses. Conventions as in panel **(E)**, except that both represent example of deep layers, and the PSTH and raster plot following blue-light application are represented in blue.

In the ChR2-transfected animals, we first analyzed cortical activity (field potentials and unitary spikes) to trains of whisker pad air-puff stimulation prior to and following a blue-light stimulation pulse applied to the tissue surrounding the recording site. To this end, we inserted an optrode at different depths within M1wk or S1BF cortex (Figure 6A). An equivalent protocol without light was delivered as control; we did not observe any change in the responses to whisker pad stimulation under this condition (*P* > 0.05).

When blue-light was applied in the superficial layers of M1wk cortex, the SEP1 recorded in these layers decreased (*F* = 3.432, *P* = 0.037; *n* = 25); however, when light was applied to the deep cortical layers, no significant changes were observed in the SEP1 recorded in these layers (*F* = 0.550, *P* = 0.580; *n* = 20; Figure 6C). In S1BF cortex, in contrast, blue-light application produced SEP1 decrease in both the superficial and deep layers (*F* = 10.141, *P* < 0.001, *n* = 15 and *F* = 3.608, *P* = 0.040, *n* = 10, respectively; Figure 6F). In some experiments as the one illustrated in Figure 6B, a small increase of the response to whisker pad stimulation in central cortical layers (around 800 μm depth, roughly corresponding to L3b-5a) was observed. These marked effects roughly matches that of the densest Po axon terminal arborizations (Figure 5; Ohno et al., 2012).

Ten seconds after we light-activated the Po axons in the superficial layers of M1wk, SEP1 area to a 1st air-puff train in the same layers went down (16.7 ± 5.2% less than control values; *P* = 0.011; *n* = 25; Figure 6C). Units recorded in the same layers likewise reduced their responsivity (from 1.0 ± 0.1 spikes/stimulus to 0.8 ± 0.1 spikes/stimulus; 24.3 ± 6.5% decrease; *P* = 0.024; *n* = 15; Figures 6D,E). In contrast, blue light applied into the deep M1wk layers did not modify significantly either the SEP1 (3.8 ± 3.7% above control values; *P* = 0.637; *n* = 20; Figure 6C) or the unit spike counts (from 1.0 ± 0.3 spikes/stimulus to 0.9 ± 0.3 spikes/stimulus; 10.1 ± 7.8% decrease; *P* = 0.915; *n* = 11; Figure 6D) elicited by air puff stimulation in these deep layers. The application of blue-light in superficial or deep M1wk layers SEP1 did no modify peak latencies to air-puff stimulation (STT, *P* = 0.787, *n* = 25 for superficial layers; STT, *P* = 0.364, *n* = 20 for deep layers; data not shown).

Blue-light activation of Po axons in S1BF significantly reduced subsequent responses in this area to whisker pad air-puff stimulation. In the superficial S1BF layers, both SEP1 and unit responses decreased (SEP1: 11.9 ± 2.4% from to control values, *P* = 0.001; *n* = 15; Figure 6F; unit responses: from 1.6 ± 0.2 spikes/stimulus to 1.4 ± 0.2 spikes/stimulus; of 13.3 ± 2.3% decrease; *P* < 0.001; *n* = 16; Figures 6G,H). The decrease was of comparable magnitude in deep S1BF layers (SEP1: 12.5 ± 4.4%, *P* = 0.031; *n* = 10; Figure 6F; unit responses: from 1.5 ± 0.3 spikes/stimulus to 1.3 ± 0.3 spikes/stimulus, 18.1 ± 4.5% decrease; *P* < 0.001; *n* = 17; Figure 6G). Lowered S1BF responsivity persisted even during the 2nd air puff train, 130–230 s after the blue-light pulse (1.3 ± 0.3 spikes/stimulus; −14.2 ± 3.7% of control train values; *P* = 0.001; *n* = 17). Similarly to M1wk, blue-light application did not modify SEP peak latencies in S1BF (STT, *P* = 0.216; *n* = 15 for superficial layers; WT, *P* = 0.249, *n* = 10 for deep layers; data not shown).

### Effect of Light-Activating Po Axon Terminals in One Layer on the Responses to Whisker Stimulation in Deeper Cortical Layers

**Figure 7:**
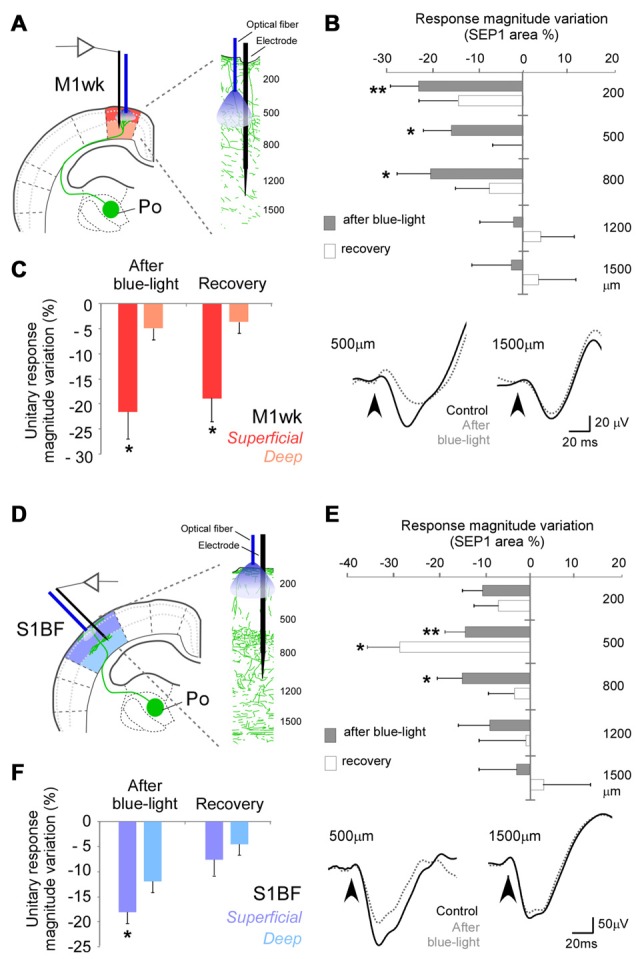
Optogenetic activations of Po terminal axons focused on M1wk layer 3 or on S1BF layer 1 modify subsequent tactile stimulus processing in other cortical layers. **(A)** Diagram of the M1wk experimental protocol. Mechanical stimuli (air puffs) were applied to the trimmed contralateral whiskers (three trains of 100 stimuli, 1 Hz). A single long-lasting pulse of blue-light (473 nm, 300 ms, <30 mW/mm^2^) was applied through a fiber optic whose tip was positioned in L3M1wk, pointing inwards to illuminate Po axons terminals expressing ChR2 + eYFP in this layer. The light pulse was applied immediately after the first train (“control”) of whisker stimuli. SEPs and unit responses were recorded separately in the superficial and deep layers (shaded in different color hues, respectively). **(B)** L3 Po terminal axons activation modified SEP1 in others M1wk cortical layers. Changes in SEP1 area immediately after light application (gray) and during the recovery train (white) are represented as the variation of percentage to the control values at different depths. Negative values symbolize inhibition and positive values facilitation. Representative traces examples of SEPs recorded during control (black line) and after light application (gray dashed line) trains are shown below. **(C)** Blue-light activation of Po terminal axons in L3 of M1wk modulates unit responses and more superficially located layers. Mean values of changes in unit responses are represented as the variation of spike counts percentage to the control train. **(D)** Diagram of the S1BF experimental protocol. A single long-lasting pulse of light was applied through a fiber optic whose tip was placed in L1 S1BF, pointing inwards. Others conventions as in panel **(A)**. **(E)** Light-activation of thalamocortical Po axons in L1 changes subsequent SEP1 recorded in others cortical layers. Conventions as in panel **(B)**. **(F)** Light activation of Po terminal axons in L1 S1BF modify unit responses in both superficial and deep layers. Conventions as in panel **(C)**.

We took advantage of the fact that Po terminals are largely confined to particular cortical layers (L2/3 in M1wk and L1 and L5a in S1BF; Figure 5C; see also Wimmer et al., 2010; Ohno et al., 2012) to investigate *in vivo* how activation of Po terminals in one of these terminal layers influences cell activity in other layers of the same cortical column. Specifically, we studied the effect of blue-light stimulation at a fixed cortical depth, on SEPs and unit responses elicited by a 100-seconds air puff train recorded at different depths using an adjacent, movable electrode (Figures 7A,D). For example, we applied blue light over the pia in S1BF to stimulate the population of Po synapses located in L1, and tested to which extent activation of these synapses in particular affect the activity of cells in deeper layers of the same column (Figures 7D–F). The same protocol without blue-light application was used as control; no change was observed in the responses to whisker pad stimulation in any layer under this condition (*P* > 0.05).

In M1wk, an optic fiber tip was lowered into the cortex at a depth of ~300 μm pointing downwards, directly illuminating L3b, where Po axons form abundant terminal arborizations (Figure 5). Blue-light application from this position (Figure 7B) markedly reduced SEP1 area elicited to subsequent whisker pad stimulation in recordings made at 200 μm (~L2, −22.5 ± 6.2%; *P* = 0.003; *n* = 15), at 500 μm (~L3b, −15.6 ± 6.0% below control values; *P* = 0.010; *n* = 12) or 800 μm under the pia surface (~L5a, −20.1 ± 4.5%, *P* = 0.024; *n* = 12). In contrast, SEP1 area remained basically unchanged in deeper recording sites (–2.1 ± 7.3% at 1200 μm, ~L6a and −2.5 ± 8.6% at 1500 μm, ~L6b, *F* = 1.205, *P* = 0.313 and *F* = 0.635, *P* = 0.536, respectively; *n* = 11 in each depth). Light stimulation did not change SEP1 peak latencies at any depth (*P* > 0.05; *n* = 15 for 200 μm, *n* = 12 for 500 and 800 μm, *n* = 11 for 1200 and 1500 μm in depth; data not shown).

Likewise, unit responses to whisker pad stimulation (Figure 7C) decreased in superficial M1wk layers (200–500 μm depth; from 1.2 ± 0.1 spikes/stimulus to 0.9 ± 0.1 spikes/stimulus, −21.6 ± 4.9% to control; *P* = 0.013; *n* = 10), and their reduction persisted even 4 min later, during the second air puff train (0.9 ± 0.1 spikes/stimulus, −10.3 ± 5.4%; *P* = 0.039). By contrast, unit responses in the deep layers did not decrease (1.2 ± 0.21 spikes/stimulus to 1.1 ± 0.2 spikes/stimulus; −4.8 ± 2.1%; *F* = 0.235, *P* = 0.792; *n* = 11) indicating that activation of Po terminal axons in L2/3 influences subsequent sensory processing in both L2/3 and L5a.

In a final set of experiments, the fiber optic placed on the pial surface of S1BF, and SEPs and unit recordings were made underneath, at different depths (Figure 7D). Blue-light illumination from this position produced significant changes in SEP1 at 200 μm (–10.2 ± 3.3%; *P* = 0.031; *n* = 7). More marked changes were observed at 500 μm (which roughly corresponds to L4 in S1BF) during the air puff train administered 10–110 s afterwards (–14.1 ± 4.4%; *P* = 0.004; *n* = 7), and even during a second air-puff train delivered 130–230 s after the light pulse (–28.4 ± 7.0%; *P* = 0.013; Figure 7E). At 800 μm, which roughly corresponds to L5a, SEP1 was also diminished (–14.7 ± 5.4%, *P* = 0.037; *n* = 7, during the first train). SEP area did not significantly change in deeper recording sites (–8.7 ± 6.8% in 1200 μm; 2.9 ± 4.4% in 1500 μm, *F* = 0.583, *P* = 0.567 and *F* = 0.078, *P* = 0.925, respectively; *n* = 7 in each case). SEP peak latencies did not change at any depth (*P* > 0.05; *n* = 7 in each cortical depth; data not shown).

Unit responses were also reduced in superficial S1BF layers (200–500 μm depth; 1.6 ± 0.34 spikes/stimulus to 1.4 ± 0.3 spikes/stimulus, −18.1 ± 2.9%; *P* = 0.001; *n* = 8; Figure 7F). As in M1wk, unit responses in deep S1BF layers were not decreased (from 2.3 ± 0.3 spikes/stimulus to 1.9 ± 0.3 spikes/stimulus, −11.9 ± 6.5%; *F* = 1.025, *P* = 0.368; *n* = 13). These results indicate that the activation of L1 Po terminal axons can decrease cortical neuron responsivity to tactile whisker pad stimulation across of S1BF cortical columns, except in L6.

## Discussion

**Figure 8:**
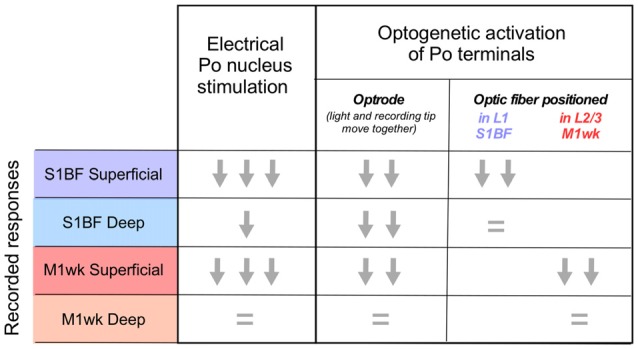
Posterior nucleus thalamocortical cell activation effects on responses to passive multi-whisker stimulation in M1wk and S1BF cortices. Graphic summary of the effects observed in the superficial and deep layers of each area in the different experimental conditions. Response magnitude is represented as one, two or three arrows. An “equal” symbol indicates no variation.

Using electrophysiology, optogenetics and pharmacological tools *in vivo*, we compared responses in the whisker areas of the motor and primary somatosensory cortices to passive multi-whisker deflection, their dependence of Po activity, and their modulation by a brief but intense train of thalamocortical Po activation. Evidence from field potentials evoked by multi-whisker stimulation before and after suppression of S1BF activity by lidocaine application, along with the effect of Po silencing with muscimol injections, indicate that the early component of M1wk responses is probably mediated by Po. Moreover, intense electrical or optogenetic activation of Po thalamocortical axons produces in both M1wk and S1BF a similar reduction of responsiveness to subsequent whisker pad tactile stimulation (Figure 8). This response reduction is most marked in the superficial cortical layers, and it might be, in part, mediated by the activity of PV-fast spiking interneurons.

### Early M1wk Responses to Tactile Stimulation Are Mediated by Po

Passive single-whisker stimulation reliably produces cell firing in S1BF and M1wk (Kleinfeld et al., 2002; Ferezou et al., 2007). *In vivo* studies with voltage-sensitive dye in mice have shown that the S1BF activation after a single-whisker stimulation consistently precedes that of M1wk by ~8 ms or more (Ferezou et al., 2007). Area S1BF receives direct “lemniscal” and “paralemniscal” trigeminal input relayed, respectively, via VPM and Po thalamic neurons. In contrast, M1wk lacks VPM lemniscal connections, and instead receives its main thalamic input from the ventrolateral nucleus, which relays cerebellar information (Kuramoto et al., 2009; Mohammed and Jain, 2014). For these reasons, it has been concluded that the delayed M1wk activation by single-whisker stimulation should be mainly mediated by cortico-cortical pathways from S1BF (Izraeli and Porter, 1995; Ferezou et al., 2007; Mao et al., 2011; Hooks et al., 2013).

It is known that M1wk receives a smaller but consistent thalamic projection from Po (Ohno et al., 2012). Since Po cells are strongly driven by S1BF layer 5b cells, the Po projection to M1wk might also contribute to the delayed M1wk activation via a polysynaptic cortico-thalamo-cortical loop (Theyel et al., 2010).

In addition, however, the Po nucleus receives paralemniscal trigeminal connections (Veinante and Deschênes, 1999; De Chazeron et al., 2004; Guy et al., 2005), and thus it might constitute a fast conduit for some sensory inputs to M1wk (Hoffer and Alloway, 2001; Ohno et al., 2012; Hooks et al., 2015). It should be pointed out that the possibility that ascending tactile inputs relayed via Po can drive directly, *per se*, M1wk cells cannot be ruled out from the delayed M1wk activation observed in the single-whisker stimulation experiments described above since single-whisker stimulation is not adequate to induce firing in thalamocortical Po cells (Diamond et al., 1992; Groh et al., 2014). Intriguingly, some previous studies that used stimuli suited to drive Po cells, such as multi-whisker deflections or direct whisker pad electrical stimulation (Farkas et al., 1999; Chakrabarti et al., 2008), observed a faster M1wk activation, beginning as soon as 2 ms after the start of the S1BF activation. Our experiments using multiple-whisker deflection consistently show that latency of the first component peak of the evoked field potential in M1wk is only marginally longer (~1 ms) than that of the peak recorded in S1BF (Figure 1C). These observations indicate that paralemniscal trigeminal information may be reaching M1wk directly via Po.

Consistent with this interpretation, we observed that silencing Po nucleus with a muscimol injection drastically reduced the SEP1 whisker responses in M1wk cortex (Figure 1E). Responses in S1BF, in contrast, were unaffected, as could be expected from the fact that the lemniscal input via the VPM nucleus remained intact (Gil et al., 1999; Bruno and Sakmann, 2006). Previous studies used the same dose and volume that we used in our experiments to silence Po, reducing Po spontaneous activity to 20% (Nakamura et al., 2009; Castejon et al., 2016; Supplementary Figure S2). Moreover, several lines of evidence indicate that at the volume and concentration we applied (0.1 μl, 1 mM), the zone of pharmacologically-effective muscimol levels in our experiments was limited to Po. The rat Po nucleus has a rostrocaudal extent of about 2.5 mm and a mediolateral width of 1.5 mm. Using tritiated muscimol (0.1 μl, 8.7 mM) and autoradiography, Edeline et al. (2002) showed that 15 min after the muscimol injection injected in the rat brain, radiation were minimal beyond 1 mm. Since in our experiments we used the same volume than Edeline et al. (2002), but almost an order of magnitude less muscimol dose, it can be safely concluded that effective levels were reached within less of a 1 mm radius around the needle tip. In fact, a recent study injecting muscimol at the same dose and volume we used (0.1 μl, 1 mM) in two nearby sites in the rat thalamus eliciting opposite behavioral effects, explicitly concluded that the effect of muscimol did not reach beyond 1 mm (Stratford and Wirtshafter, 2013). As a final confirmation, we conducted control experiments that directly showed that, at the concentration and volume applied, muscimol injections in Po do not modify the excitability of the rostrally adjacent ventrolateral nucleus (Supplementary Figure S2).

Regarding the effectivity of the dose applied, in addition to the studies just cited above, Castejon et al. (2016) using the same muscimol dose and delivery method showed that a VPM muscimol injection drastically reduces tactile-evoked responses in somatosensory cortex, in a manner comparable with the effect of lesioning this nucleus (see Yang et al., 2013). At the same time, in our study, we did not find a reduction in S1BF area of somatosensory evoked potentials. Since the barreloid domains of VP are located just at the border of VPM with Po, the above observation indicates that VPM nucleus was not affected. Future studies using selectively targeted/rapidly reversible optogenetic or pharmacogenetic activity blocking tools may confirm and add further insight on the effect of silencing Po on sensory and motor function *in vivo*.

In a recent study of our group (Castejon et al., 2016), we observed that muscimol injections in Po increase the whisker-evoked firing rate of S1BF neurons. The apparent contrast with the findings reported here likely reflects the different methodologies used for each study. In Castejon et al., unit supra-threshold responses were recorded. In the present study, we did not record unit responses, but the averaged evoked potentials of hundreds of cells near the electrode in S1BF, which include both supra- and subthreshold membrane activity. Increased firing of some S1BF cells to whisker stimulation was thus most likely masked in the averaged field potentials.

Also consistent with the possibility that whisker pad stimulation directly activates M1wk cells via Po, we observed that S1BF silencing with lidocaine did not reduce the first component of the evoked potential in M1wk (SEP1, Figure 1D). Two previous studies that examined the effect of S1BF lidocaine inactivation also did not find consistent changes in M1wk responses (Izraeli and Porter, 1993; Farkas et al., 1999). Farkas et al. (1999) reported that aspiration of the whole S1 cortex abolished M1wk responses; the significance of this observation, however, is unclear because the Po axons that innervate M1wk travel directly under S1BF and even through S1BF deep layers (Deschênes et al., 1998; Ohno et al., 2012), thus making it likely that aspiration may have significantly damaged/destroyed Po connections to M1wk.

In addition to the responses to tactile whisker pad stimulation, we found that *in vivo* electrical activation of Po nucleus induced orthodromic spikes in M1wk cells (Figure 4B). In both cases, the responses were larger and have shorter peak latencies in the superficial layers (L2-L4) than in deep layers (L5-L6), consistent with the distribution of Po axons terminal arborizations (Figure 5E; Deschênes et al., 1998; Ohno et al., 2012; Hooks et al., 2015).

Overall, the evidence from the above experiments indicates that Po may act as direct relay for paralemniscal inputs able to drive M1wk cells, particularly those in the superficial cortical layers.

### Electrical or Optogenetic Activation of Po Axons Reduces M1wk and S1BF Responsiveness to Subsequent Tactile Stimulation

High-frequency electrical stimulation of the VPM projections has been shown to reduce subsequent whisker-evoked cortical responses in S1BF (Middleton et al., 2010). Whether Po projections are able to modulate in a similar way the responsiveness to tactile stimulation of S1BF or M1wk cells had not been investigated. Here, we show that, *in vivo*, both a brief (1 s) electrical stimulation of Po or a light-pulse stimulation (300 ms) of optogenetically transfected thalamocortical Po terminals lessens the responsiveness of cortical cells to subsequent tactile whisker pad stimulation for several minutes (see Figure 8).

With both stimulation protocols, inhibition of responsiveness in M1wk was most marked and prolonged in the superficial cortical layers (Figures 2C, 6C,D). This laminar preference may be related to the fact that in rodents most Po synapses in M1wk are located in L2-L4 (Wimmer et al., 2010; Ohno et al., 2012). In S1BF, the responsiveness decrease was significant and lasting in both the superficial and deep cortical layers (Figures 2D, 6E,F). Again, this pattern might bear relation to the fact that Po axons in S1BF terminate preferentially in L5a and L1 (Lu and Lin, 1993; Ohno et al., 2012). A recent study using electrical Po activation similarly and testing shorter-term effects observed a general inhibitory modulation of the S1BF unit responses to whisker pad tactile stimulation (Castejon et al., 2016). A study using *in vivo* optogenetic stimulation of Po terminals in S1BF has recently reported an increase of responsiveness in upper layer 5 units (Mease et al., 2016). This finding might correspond to the small but consistent increase in response observed in our recordings at around 800 μm in S1BF (Figure 6B).

The effects of Po activation on cortical cell responsiveness over even longer time expanses (tens of minutes) have been recently investigated *in vitro* (Gambino et al., 2014; Maglio et al., 2017). Gambino et al. (2014) have shown that whisker stimuli recruit synaptic networks that originate from Po, generating NMDAR-mediated plateau potentials that are involved in whisker-evoked LTP. These studies have shown that strong activation of Po monosynaptically modulates subsequent S1BF cell responsiveness and plasticity for relatively long periods.

### Lowered M1wk Responsiveness Following Po Activation Might be Mediated by PV Interneurons

Depression of responsiveness following thalamocortical activation is believed to involve both a history-dependent depression of thalamocortical synapses and an engagement of cortical inhibitory neurons (Castro-Alamancos and Oldford, 2002; Khatri et al., 2004; Middleton et al., 2010). Fast-spiking PV-expressing GABAergic interneurons control cortical excitability and are a major synaptic target of thalamocortical axons (Cruikshank et al., 2007; Courtin et al., 2013; Zhang et al., 2015). Electron microscopic studies have shown that a substantial proportion of thalamocortical synapses in L2/3 of rat frontal cortices are in fact established on both somata and proximal dendrites of the fast-spiking PV-expressing interneurons (Kawaguchi and Kubota, 1997; Shigematsu et al., 2016).

Fast spiking PV-expression generate the di-synaptic feedforward inhibition characteristic of thalamocortical transmission (Castro-Alamancos and Connors, 1997). To dissect the possible involvement of PV interneurons in the lasting SEP1 decrease to whisker pad stimulation observed in M1wk after a brief electric activation of Po, we interfered with GABA release from the synapses of these interneurons by intracortically injecting omega-agatoxin (Figures 3B–D; Toledo-Rodriguez et al., 2004; Hefft and Jonas, 2005; Zaitsev et al., 2007; Rossignol et al., 2013). Omega-agatoxin is a blocker of Cav2.1 P/Q channels and these channels are expressed abundantly in the GABAergic synapses of fast spiking PV interneurons (Toledo-Rodriguez et al., 2004; Hefft and Jonas, 2005; Zaitsev et al., 2007). Others have shown that the release of GABA in these interneurons involves N-type Ca^2+^ channels at one type of synapse and P-type Ca^2+^ at another (Poncer et al., 1997). Omega-agatoxin significantly reduces fast spiking interneurons excitability and GABA release at their synapses (Ali and Nelson, 2006). The decreased cortical inhibition we observed seems consistent with a suppression of normal fast spiking PV interneuron GABA release by omega-agatoxin.

The interpretation of the effect of omega-agatoxin in the cortex, however, is complicated by the fact that Cav2.1 P/Q channels are also expressed in some glutamatergic neurons. Thus, on the one hand, in the pyramidal cell synapses onto the fast spiking interneurons, normal Cav2.1 P/Q channel activity favors enhanced recruitment of these interneurons and increased inhibition (Pietrobon, 2005; Tottene et al., 2009). It has been shown that the Cav2.1 P/Q channels are preferentially located in a population of pyramidal cell-FS synapses that displays synaptic depression (Ali and Nelson, 2006). On the other hand, the Cav2.1 P/Q channels may also be present in glutamatergic terminals between pyramidal cells the favoring and increased network excitation. Suppression of this activity would, in principle, favor lesser cortical excitability.

Since, under our experimental conditions, omega-agatoxin application consistently increased cortical responsivity to Po activation; it is likely that the toxin effect on GABA release by fast spiking interneurons predominated overall. We favor this conclusion because: (i) the amplitude of whisker-evoked cortical potentials *increased*; and (ii) the Po-mediated inhibition that we normally observed in the cortex *decreased* after the toxin application (Figure 3).

Additional indication in favor of the participation of PV-expressing interneurons is provided by our confocal microscopic observation of frequent appositions between PV-immunolabeled cells with terminal varicosities of ChR2-eYFP transfected Po axons in brain sections of the animals recorded in the optogenetic assays (Supplementary Figure S3). Although further electron microscopy analysis would be necessary to ascertain the existence of actual synapses, the observed abundance of these contacts is consistent with the electron-microscopic literature (Kawaguchi and Kubota, 1997; Shigematsu et al., 2016).

## Concluding Remarks

Whisking rodents infer the position, size and texture of objects around their head by computing relationships between motor commands to whisker pad muscles and mechanoreceptive signals from the whisker pad. These computations are carried out by multi-level neuronal loops including thalamic nuclei VPM and Po and cortical areas M1wk and S1BF (Ahissar and Zacksenhouse, 2001; Yu et al., 2006, 2015; Mao et al., 2011; reviewed in Bosman et al., 2011). In the course of exploration, M1wk and S1BF cortex actively direct rhythmic whisker movements (Matyas et al., 2010; Ahissar and Oram, 2015). Pyramidal neurons in L5 of both these areas innervating the brainstem motor centers send collateral axonal branches to Po, where they may exert a powerful effect (Urbain and Deschênes, 2007; Theyel et al., 2010; Groh et al., 2014). Po also receives direct ascending somatosensory information from the spinal and principal trigeminal nucleus (Veinante and Deschênes, 1999; De Chazeron et al., 2004; Guy et al., 2005; present results). Thalamocortical Po cells may thus convey back to M1wk and S1BF the product of dynamic computations between motor commands vs. ascending sensory signals (Groh et al., 2014; Ahissar and Oram, 2015). The observed ability of Po activity to fine-tune cortex excitability suggests that its signals might participate in the cortical mechanisms that discriminate unpredicted signals from system “noise” during whisking.

Besides, it is interesting to consider that M1wk and S1BF are not only cortico-cortically connected to each other (Mao et al., 2011) but also trans-thalamically linked via Po (Ohno et al., 2012). Similar transthalamic loops have been repeatedly found between separate cortical domains linked by dense reciprocal connections (Mesulam, 2009). Cortico-thalamo-cortical loops might be crucial for the functional coordination of slow (Steriade et al., 1993) and fast (Llinás et al., 1998) cortical oscillations in these domains during cognitive events (Jones, 2001; Saalmann et al., 2012; Nakajima and Halassa, 2017; Schmitt et al., 2017). In this context, our findings would register with the notion that Po activity may enhance the functional connectivity between separate cortical areas involved in whisking, a basic cognitive task in rodents.

## Author Contributions

DC-T and ÁN designed the experiments, performed the experiments and analyzed the data; DC-T, FC and ÁN prepared the figures and wrote the article.

## Conflict of Interest Statement

The authors declare that the research was conducted in the absence of any commercial or financial relationships that could be construed as a potential conflict of interest.

## Supplementary Material

FIGURE S1 Effect of muscimol application in Po and ventral lateral (VL) thalamic nucleus responses. **(A)** Muscimol injection into Po nucleus decreases responses in the same nucleus. Representative SEP1 trace recorded in Po nucleus to whisker pad stimulation (20 ms duration; 1 Hz; 100 stimuli). Responses in control condition (before; gray dashed line) and after muscimol injection (0.1 μl; 1 mM) into Po. Note that whiskers-evoked responses decreased 10 and 20 min (blue and purple lines) after muscimol injection into Po. **(B)** Responses in the ventral lateral thalamic nucleus are not modified by muscimol injection in Po nucleus. Representative case traces of VL responses to M1 cortex electrical stimulation (0.1 ms; 20–50 μA; 100 stimuli) before; (gray dashed line) and after muscimol injection (0.1 μl; 1 mM) into Po (colored lines). Note that responses were not decreased by the muscimol injection.

FIGURE S2 Po cell populations transfected with ChR2-eYFP viral vector. **(A)** Camera-lucida reconstruction of the complete extent of the transfected cell population (green area) in the case photographed in Figure 5. **(B–E)** Images from the center of the transfected cell population as seen on coronal sections lightly counterstained with thionin. Distance to bregma is indicated in the right upper corner. Bars: **A** = 1 mm, **B–E** = 500 μm. Additional abbreviations: LP, Lateral posterior thalamic nucleus; LPMR, Mediorostral part of LP nucleus; LPLR, Laterorostral part of LP nucleus; DLG, dorsal lateral geniculate nucleus; PF, Parafascicular thalamic nucleus.

FIGURE S3 Transfected Po axon varicosities form frequent close appositions with parvalbumin (PV)-expressing interneurons. High-resolution 3D-confocal analysis of double-labeling in L3 of M1wk. The eYFP fluorescence image (green) and PV immunofluorescence image (red) are overlaid. For each field, three orthogonal views (a direct image and two Z-stack reconstructions, not corrected for shrinkage) are illustrated. **(A–C)** Three appositions (arrowhead) between an eYFP-expressing Po axon varicosity and a PV-immunostained interneuron soma. **(D–F)** Three appositions (arrowhead) between an eYFP-expressing Po axon varicosity and PV-immunostained dendrites. A higher-magnification detail of the apposition is shown in the lower left corner insets. Bars: **A–F** = 5 μm; **D–F** Insets = 1 μm.
