# Supplementary figures and images for: Posterior Thalamic Nucleus Modulation of Tactile Stimuli Processing in Rat Motor and Primary Somatosensory Cortices
Source: Front Neural Circuits. 2017 Sep 27;11:69. doi: 10.3389/fncir.2017.00069 (PMC5623691; doi:10.3389/fncir.2017.00069)

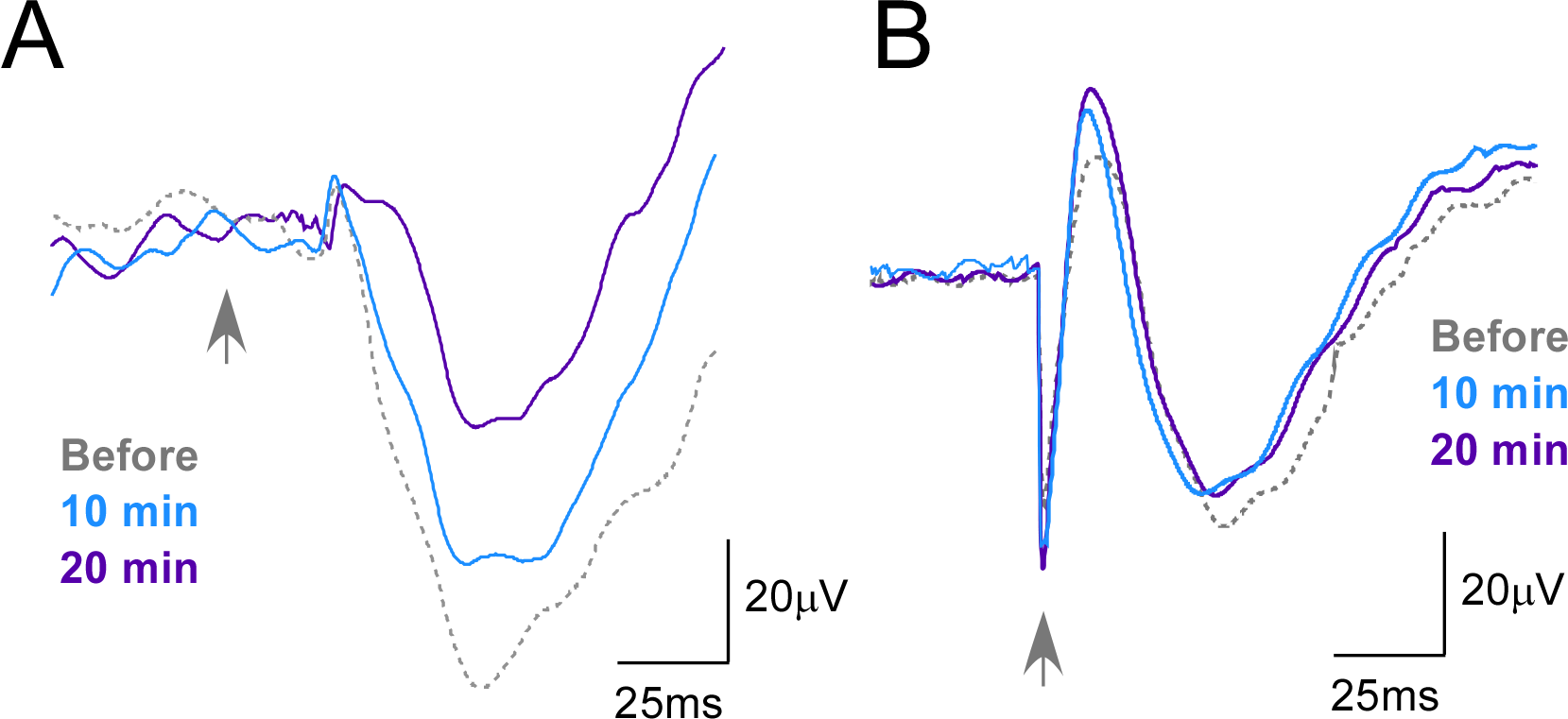

Supplement: FIGURE S1 — Effect of muscimol application in Po and ventral lateral (VL) thalamic nucleus responses. (A) Muscimol injection into Po nucleus decreases responses in the same nucleus. Representative SEP1 trace recorded in Po nucleus to whisker pad stimulation (20 ms duration; 1 Hz; 100 stimuli). Responses in control condition (before; gray dashed line) and after muscimol injection (0.1 μl; 1 mM) into Po. Note that whiskers-evoked responses decreased 10 and 20 min (blue and purple lines) after muscimol injection into Po. (B) Responses in the ventral lateral thalamic nucleus are not modified by muscimol injection in Po nucleus. Representative case traces of VL responses to M1 cortex electrical stimulation (0.1 ms; 20–50 μA; 100 stimuli) before; (gray dashed line) and after muscimol injection (0.1 μl; 1 mM) into Po (colored lines). Note that responses were not decreased by the muscimol injection. [file Image_1.tif]

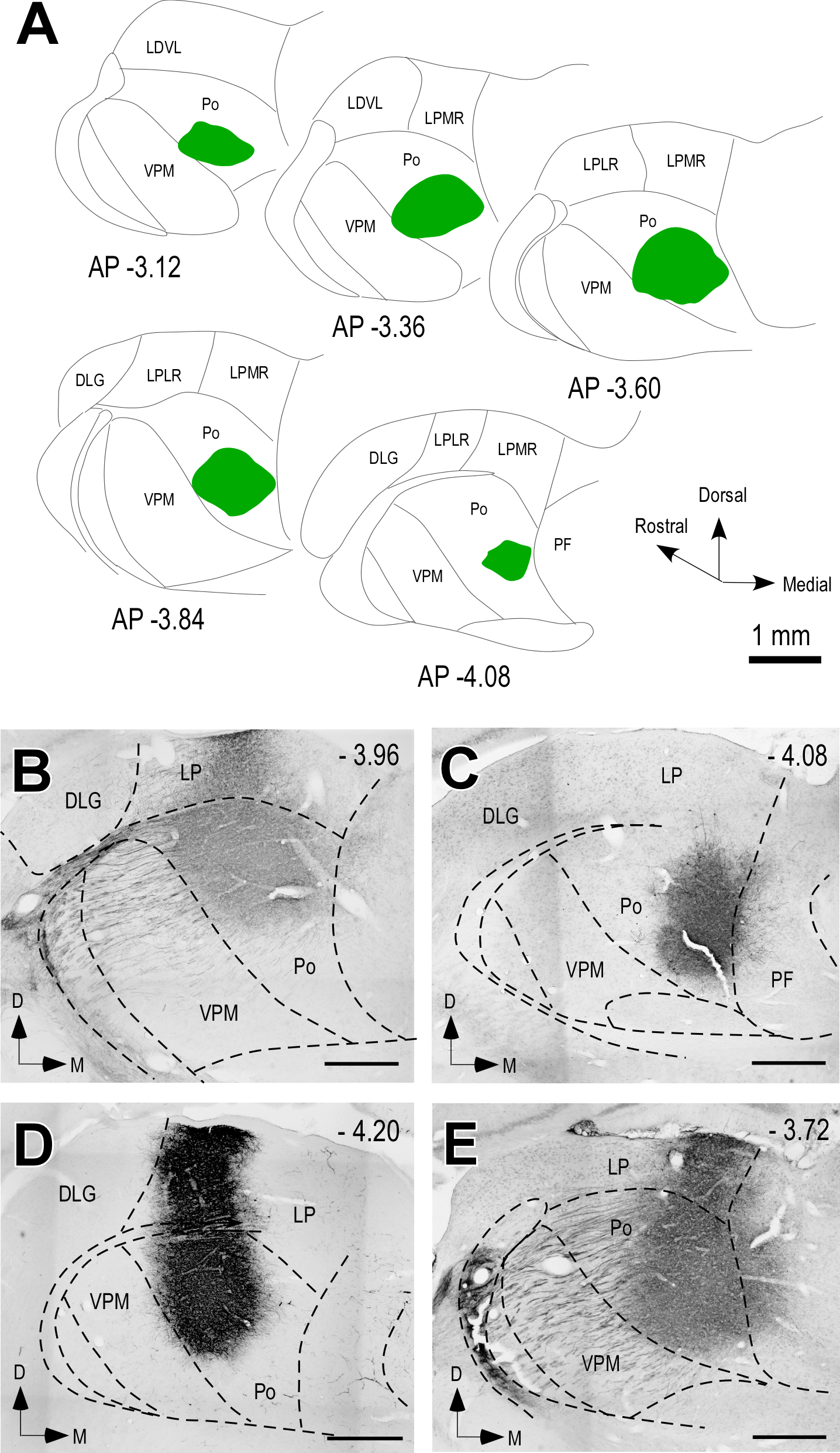

Supplement: FIGURE S2 — Po cell populations transfected with ChR2-eYFP viral vector. (A) Camera-lucida reconstruction of the complete extent of the transfected cell population (green area) in the case photographed in Figure 5. (B–E) Images from the center of the transfected cell population as seen on coronal sections lightly counterstained with thionin. Distance to bregma is indicated in the right upper corner. Bars: A = 1 mm, B–E = 500 μm. Additional abbreviations: LP, Lateral posterior thalamic nucleus; LPMR, Mediorostral part of LP nucleus; LPLR, Laterorostral part of LP nucleus; DLG, dorsal lateral geniculate nucleus; PF, Parafascicular thalamic nucleus. [file Image_2.tif]

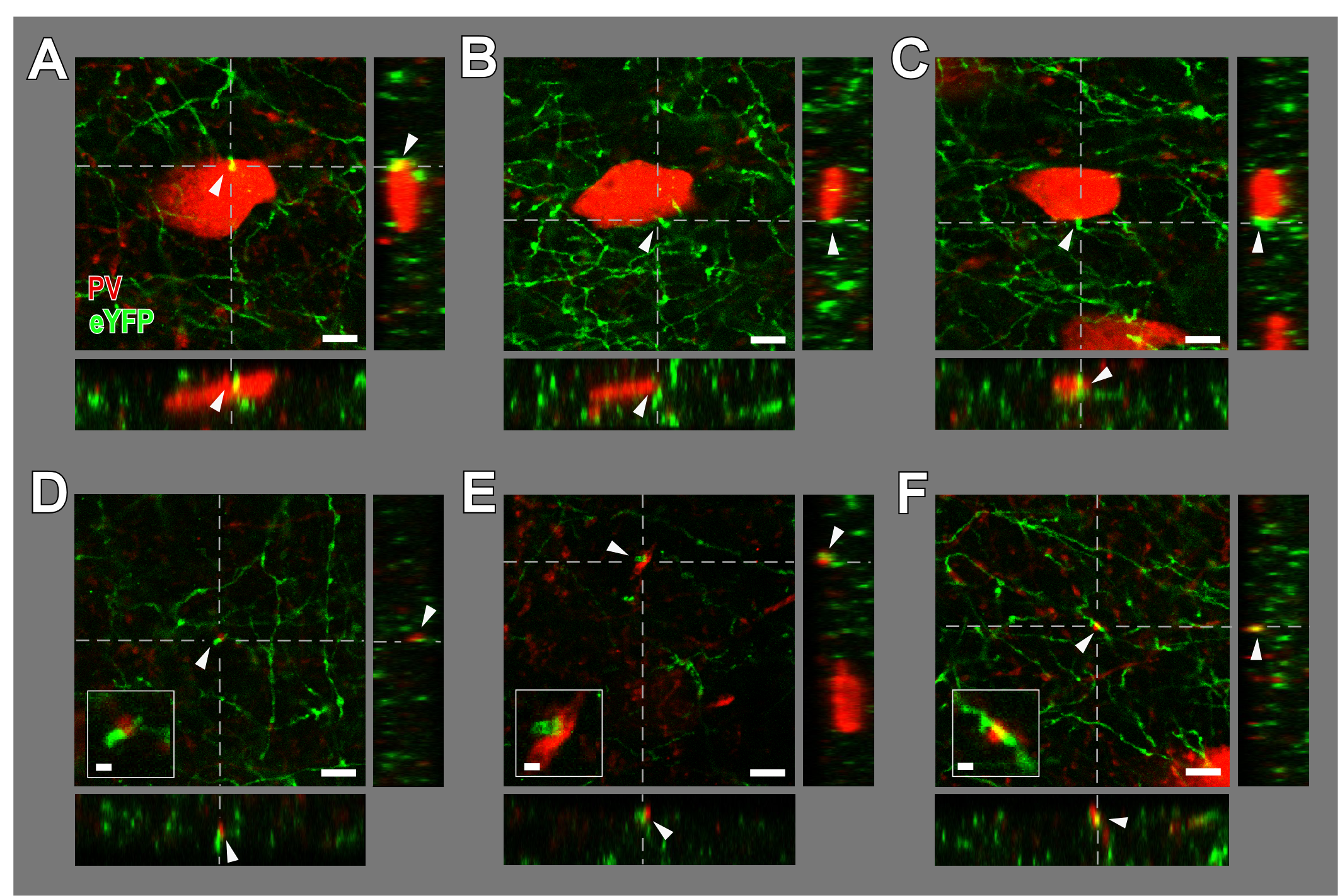

Supplement: FIGURE S3 — Transfected Po axon varicosities form frequent close appositions with parvalbumin (PV)-expressing interneurons. High-resolution 3D-confocal analysis of double-labeling in L3 of M1wk. The eYFP fluorescence image (green) and PV immunofluorescence image (red) are overlaid. For each field, three orthogonal views (a direct image and two Z-stack reconstructions, not corrected for shrinkage) are illustrated. (A–C) Three appositions (arrowhead) between an eYFP-expressing Po axon varicosity and a PV-immunostained interneuron soma. (D–F) Three appositions (arrowhead) between an eYFP-expressing Po axon varicosity and PV-immunostained dendrites. A higher-magnification detail of the apposition is shown in the lower left corner insets. Bars: A–F = 5 μm; D–F Insets = 1 μm. [file Image_3.tif]
